# Integrative structural interactomics reveals protein organization and structure in a giant virus

**DOI:** 10.1038/s41467-026-74973-2

**Published:** 2026-07-13

**Authors:** Lars Mühlberg, Julia Ruta, Vasilii Mikirtumov, Raymond Burton-Smith, Kazuyoshi Murata, Mikhail Kudryashev, Kenta Okamoto, Boris Bogdanow, Fan Liu

**Affiliations:** 1 Department of Structural Biology, Leibniz-Forschungsinstitut für Molekulare Pharmakologie, Berlin, Germany; 2 Charité Universitätsmedizin Berlin, Berlin, Germany; 3 In situ Structural Biology, Max Delbrück Center for Molecular Medicine in the Helmholtz Association (MDC), Berlin, Germany; 4 Institute of Chemistry and Biochemistry, Freie Universität Berlin, Berlin, Germany; 5 Institute of Medical Physics and Biophysics, Charité Universitätsmedizin Berlin, Berlin, Germany; 6 National Institute for Physiological Sciences (NIPS), National Institute of Natural Sciences (NINS), Okazaki, Japan; 7 Exploratory Research Center on Life and Living System (ExCELLS), National Institutes of Natural Sciences (NINS), Okazaki, Japan; 8 Department of Physiological Sciences, The Graduate University for Advanced Studies (SOKENDAI), Okazaki, Japan; 9 Laboratory of Molecular Biophysics, Department of Cell and Molecular Biology, Uppsala University, Uppsala, Sweden; 10 Institute of Virology, Charité Universitätsmedizin Berlin, Freie Universität Berlin and Humboldt-Universität zu Berlin, Berlin, Germany

**Keywords:** Virology, Protein-protein interaction networks, Structural biology

## Abstract

Giant viruses are large DNA viruses that infect unicellular and multicellular eukaryotes and form exceptionally large extracellular particles. (Meta)genomics and (meta)transcriptomics have provided insight into their diverse coding repertoire, but many of the proteins remain to be characterized as they lack homology with known proteins. Here, we integrate cross-linking mass spectrometry, quantitative proteomics, computational tools and cryo-EM data to characterize the protein architecture of intact melbournevirus particles. Based on this, we allocate 88 viral proteins to different virion sub-compartments and propose topologies of 25 inner membrane proteins. We assign eight components of the capsid in cryo-EM data, including proteins that tether the capsid shell to the membrane, reflecting key points in virion maturation. The data provide a valuable resource and demonstrate the power of an integrative approach to gain system-level structural insights into a poorly characterized biological system.

## Abstract (web summary)

Giant viruses challenge the conventional distinction between viruses and unicellular life due to their large size, genetic complexity, and proteome content. Here, the researchers used integrative structural proteomics to characterize virion proteins in melbournevirus, a Marseilleviridae member.

## Introduction

Advances in (meta)genomics and (meta)transcriptomics have revealed the remarkable genetic diversity of viruses, the largest on earth^1–3^. More recently, breakthroughs in protein structure prediction, such as Alphafold2/3, have enabled large-scale modeling of viral protein structures^4,5^. However, approximately 40% of the viral proteins lack known structural homologs^6^. Moreover, the performance and accuracy of AlphaFold’s predictions depend heavily on the availability of sequence data^7^, which is limited for many viruses, especially emerging ones^8^. These challenges hinder our ability to infer protein structures and functions solely through in silico analyses, underscoring the need for complementary experimental approaches to study poorly characterized viruses and their interactions with the host.

Giant viruses are a prime example of complex yet poorly understood biological systems. As members of the nucleocytoplasmic large DNA viruses (NCLDV), they infect unicellular and multicellular eukaryotes and produce very large extracellular viral particles^9^. Their particle size (ranging from 200 nm to up to 2 µm) and genome content (up to 2.8 megabase pairs) challenge conventional definitions of viruses^10–12^. Intriguingly, giant viruses encode proteins involved in processes once thought exclusive to cellular life, such as metabolism, protein synthesis, or genome condensation^13^. For example, the giant melbournevirus of the *Marseilleviridae* family, first described in 2014, encodes three histone-like proteins (MEL_368, MEL_369, and MEL_149) that compact its viral DNA in nucleosome-like structures - a process previously associated primarily with eukaryotes^14,15^. This genome packaging mechanism appears to be unique to members of the *Marseilleviridae* and *Medusaviridae* families^14,16,17^ because other NCLDVs, such as mimivirus assemble a large filamentous DNA-protein complex instead^18^. However, how melbournevirus genome packaging as well as transcription initiation are supported by interactions of other viral proteins with the histone-like proteins remains unclear.

The melbournevirus genome is highly complex, 360 kbp-long and contains 403 predicted open reading frames (ORFs)^19^. The virus replicates and assembles in amoeba *Acanthamoeba castellanii*, and newly formed virions exit the host cell via exocytosis or cell lysis^19–22^. Virus replication takes place in specifically induced cytosolic compartments called viral factories, which also recruit host nuclear factors for early transcription. Therefore *Marseilleviridae* virions lack a virus-encoded transcription machinery^23,24^.

Despite its significance, the structure of melbournevirus remains poorly characterized. The particle has a diameter of 230 nm and consists of several components: (1) an outer capsid shell^25^, formed by homotrimeric major capsid protein (MCP, MEL_305), along with penton and cap proteins^26^, (2) an internal membrane layer enclosing the viral DNA genome as well as an unidentified large dense structure adjacent to the membrane^25^, and (3) a minor capsid protein (mCP) layer beneath the MCP layer, likely stabilizing the capsid structure and linking it to the inner membrane^27–30^. This stabilizing linkage is highly relevant for the unique and divergent giant pseudo-icosahedral particle assembly strategies of NCLDVs^31,32^. In melbournevirus, the mCP layer’s supporting and regulating function is particularly important because no tape measure protein has been identified for the capsid, which is often observed in other giant viruses^26^.

The architecture of identified protein complexes has been determined exclusively by cryo electron microscopy (cryo-EM)^25–29^. However, the large size of capsid imposes resolution limitations that can be achieved in cryo-EM studies^27^. Due to these constraints and the complexity of the system, only four melbournevirus proteins have been structurally characterized: the major capsid protein MEL_305, and three nucleosome-like proteins MEL_368, MEL_369 and MEL_149^14,17,33^. Additionally, many melbournevirus ORFs are predicted to encode proteins lacking available homology to any known proteins in databases^10^, severely constraining our understanding of the identity, structure, function, interactions, and spatial organization of the viral proteins in the virion. These challenges could potentially be addressed by analyzing the virion’s native protein interaction networks. Cross-linking mass spectrometry (XL-MS) is particularly well-suited for this task, as it enables the mapping of protein interaction networks within intact cells^34^, organelles^35^ and virions^36^. Furthermore, integrating XL-MS with computational protein structural prediction offers a powerful approach to validate and refine structural models^37–39^.

In this study, we combine proteome-wide XL-MS with AlphaFold3-based structure prediction to characterize the structural interactome of intact melbournevirus particles. Our XL-MS data enable spatial mapping of 88 viral proteins to the two virion sublayers. Notably, within the membrane separating these two sublayers, we identify 25 viral transmembrane (TM) proteins and determine their topologies. Curiously, most viral TM proteins position the majority of their non-TM domains within the capsid-membrane space. By combining XL-MS, AlphaFold3 and cryo-EM data, we demonstrate that two of these TM proteins structurally bridge the inner virion membrane and the capsid, functioning as minor capsid proteins (mCPs). Furthermore, we generate structural predictions for multiple uncharacterized viral proteins, propose molecular functions based on their structure, localization and interaction partners and assign previously unresolved cryo-EM densities. Our findings provide detailed structural insight into melbournevirus protein organization and establish integrative structural interactomics as a powerful approach for multi-level analysis of giant viruses. More broadly, this methodology represents an attractive strategy for studying poorly understood biological systems.

## Results

### The melbournevirus interactome clusters packaged proteins to sub-virion compartments

**Fig. 1:**
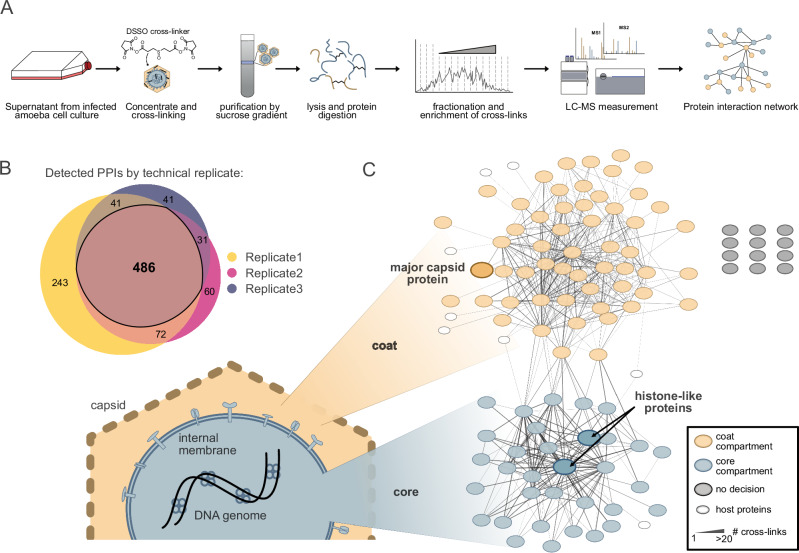
Protein interactome profiling of melbournevirus by XL-MS. **A** Workflow for XL-MS-based PPI profiling of intact melbournevirus particles. **B** Venn diagram of PPIs detected in three biological replicates. PPIs were aggregated from cross-linked peptide pairs filtered at 1% FDR. **C** XL-MS-based interaction network of melbournevirus. Proteins are categorized by localization: coat (external to the membrane, yellow), and core (internal to the membrane, blue). Proteins with only intra-protein cross-links are colored gray. The final dataset comprised 8,908 cross-linked peptide pairs, with PPIs retained only if detected in all replicates. Line thickness corresponds to the number of cross-links supporting each PPI. Network was created using Cytoscape^76^. The network with annotated gene names is shown in Supplementary Fig. 1. Source Data are provided.

We employed XL-MS to uncover protein-protein interactions (PPIs) within the large and poorly characterized melbournevirus particles. Virions were collected from the supernatant of infected *A. castellanii* cell culture and cross-linked using the MS-cleavable cross-linker disuccinimidyl sulfoxide (DSSO). To enrich virions and deplete contaminating debris, we purified the particles by sucrose density-gradient ultracentrifugation, as described previously^19,25^. Proteins were then extracted, digested by trypsin, and cross-linked peptides were identified by LC-MS (Fig. 1A).

To assess the reproducibility of our approach, we performed XL-MS across three biological replicates. We considered lysine, serine, threonine, and tyrosine as possible DSSO reaction sites, with 92% of all identified cross-links being lysine-lysine pairs. We identified between 4900 and 8100 unique residue-to-residue connections at a residue-level false discovery rate (FDR) of 1% (Supplementary Data 1). After aggregating cross-links to PPIs, we analyzed the overlap between replicates. Approximately 64% of PPIs were identified in at least two replicates, and 50% were present in all three, indicating high experimental reproducibility (Fig. 1B). To minimize false positives, we retained only PPIs observed in all three replicates. The resulting filtered interaction network comprised 486 PPIs, corresponding to 8,908 cross-links from 108 proteins (Fig. 1C and Supplementary Fig. 1, Supplementary Data 1).

While most cross-links involved viral proteins, a small fraction (2–4.5% per replicate) connected viral and host proteins. We included only those amoeba host proteins with direct viral interaction partners, likely representing virion-recruited host proteins. These included amoebal Cystatin, an HMG (High mobility group) box domain-containing protein, Peptidyl-prolyl cis-trans isomerase, Vinculin, and several unannotated proteins corresponding to previously identified transcripts in amoeba^40^. The predominance of viral proteins and the scarcity of highly abundant host proteins inside the virion align with prior proteomic analyses^23^.

Next, we investigated the spatial distribution of packaged proteins. Previous cryo-EM studies revealed that melbournevirus possesses a 30 Å-thick inner membrane layer separating the outer capsid from the inner dsDNA genome and associated proteins^25^. This membrane imposes a constraint on XL-MS, as the DSSO cross-linker, which has a spacer arm of 10.1 Å, cannot connect proteins on opposite sides. To evaluate this constraint in our PPI network, we performed unsupervised community clustering^41^, which partitioned the network into two major protein communities (Supplementary Data 2). Proteins within the same community exhibited dense interactions, whereas connections between communities were sparse. These communities reflect the spatial segregation of a genome-associated internal core and a membrane-external, capsid-enclosed coat.

We then predicted sub-virion localizations for melbournevirus proteins based on the only four proteins with known localizations: the histone-like viral proteins MEL_368 [H4-H3-analog], MEL_369 [H2B-H2A-analog] and MEL_149 [“mini” H2B-H2A-analog]^14^ localized to the core, while the major capsid protein (MCP, MEL_305) resided in the coat^25,26^. Using the spatial information of their interaction partners, we classified the viral proteome into 33 core and 55 coat proteins. Thus, based on protein connectivity, we derive sub-virion localization of a substantial part of the virion proteome.

### Transmembrane predictions and cross-linking data assign topologies to viral TM proteins

**Fig. 2:**
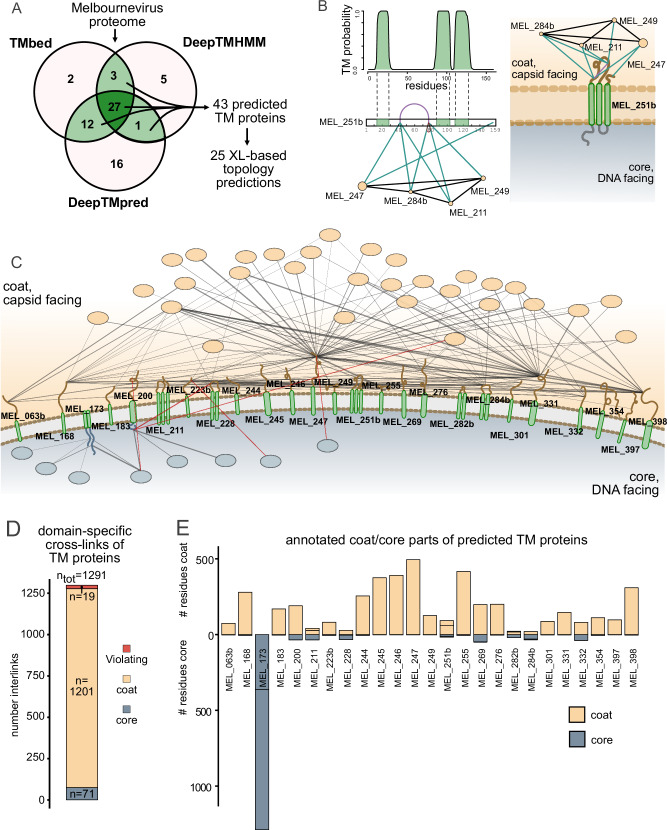
Topology analysis of virion-incorporated transmembrane proteins in melbournevirus. **A** Venn diagram of the TM proteins predicted by three different algorithms within the melbournevirus proteome. Only TM proteins with detected intra-protein cross-links were included in further analysis. **B** Representative example of topology annotation, combining predicted TM domains (green) and cross-linking data. Regions that cannot be predicted (e.g., lacking cross-links) are shown in gray. Interacting proteins are color-coded by predicted localization as in Fig. 1. Mel_251b is shown as a horizontal bar representing the protein from N- to C-terminus and residue-specific cross-links are shown using XiNet^77^. **C** Overview of predicted TM protein topologies and their direct interaction partners. Cross-links within the same domain are grouped for clarity and gene names of interactors from coat and core compartments are removed. Cross-links supporting the proposed topology are shown in black; those violating the topology are highlighted in red. Network was created using Cytoscape^76^. A fully annotated network is shown in Supplementary Fig. 2. **D** Number of cross-links in the three different categories shown in (**C**). **E** Number of amino acids in the coat and the core of the 25 predicted TM proteins where domains could be annotated. In case of multiple domains per protein, the domains in the same compartment are added onto another as indicated by black lines separating those parts. Source Data are provided.

Next, we sought to identify proteins bridging the two communities, focusing on membrane-anchored proteins in our cross-linking dataset. Since no experimental data were available for transmembrane (TM) domain annotation of melbournevirus, we screened the viral proteome using in silico prediction tools (DeepTMHMM, DeepTMpred, and TMbed; Supplementary Data 3). These algorithms predict both α-helical and β-barrel TM regions with high accuracy^42–45^. To enhance reliability, we retained only TM domains predicted by at least two algorithms. While TMbed and DeepTMHMM showed strong agreement, DeepTMpred assigned more TM domains (Fig. 2A), likely because it does not distinguish between TM domains and signal peptides^43,45^. This analysis identified 43 putative TM proteins, of which 25 were present in our interaction network (Fig. 2A).

A key advantage of XL-MS is its domain-level resolution, enabling topology prediction of TM proteins^35^. Leveraging this, we assigned topologies based on the localizations of interacting partners in the PPI network (Fig. 2B). Using 1291 cross-links, we predicted topological orientations for 29 domains of 25 predicted TM proteins (Fig. 2C and Supplementary Fig. 2, Supplementary Data 3). These predictions explained 98.5% of the cross-linking data (Fig. 2D). The remaining ~ 1.5% of cross-links conflicted with our predictions, possibly due to mis-incorporated proteins, false identifications or proteins with dual topologies^46^.

Notably, most cross-links on TM proteins mapped to the outer membrane leaflet. Consistently, non-TM domains were predominantly located in the coat (Fig. 2E). This suggests melbournevirus TM proteins evolved with large coat-facing domains and small core-facing regions. One exception is MEL_173, which has two non-TM domains, both predicted in the core. Taken together, our XL-based analysis resolved a high-resolution sub-virion interactome, revealing dozens of viral TM proteins with a preferential outer-leaflet exposure.

### Cross-links validate structure prediction of melbournevirus proteins

**Fig. 3:**
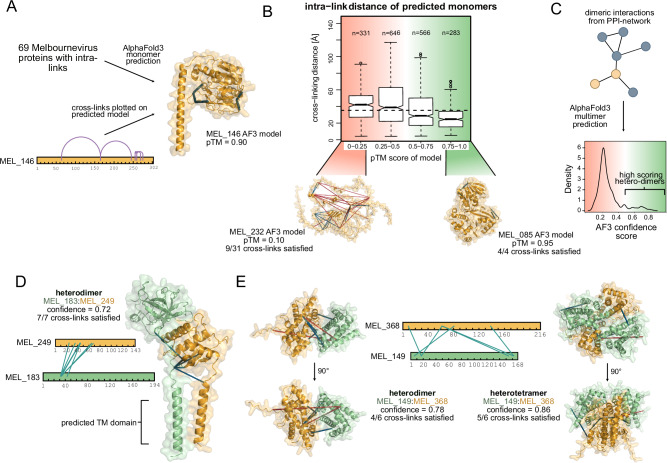
AlphaFold3-based structure prediction and cross-linking validation. **A** AlphaFold3-predicted structures of 58 Melbourneviral proteins with intra-protein cross-links, evaluated using a 35 Å maximum distance constraint. Representative example (MEL_146) shows cross-link mapping (blue lines) on the predicted structure. **B** Agreement between cross-link-derived distances (Cα–Cα) and model confidence (pTM scores; very low, 0.2–0.4; low, 0.4–0.6; medium, 0.6–0.8; high 0.8–1.0). Higher-confidence models better satisfy the 35 Å constraint than low-confidence predictions. Boxes indicate the lower and upper quartiles, with median shown as a horizontal line. The whiskers extend to 1.5 times the interquartile range. **C** Heterodimeric structural prediction of cross-linked protein pairs by AlphaFold3. Density plot of AlphaFold composite confidence scores (see “Methods”) reveals limited high-confidence predictions (right-skewed distribution). **D** Heteromultimeric prediction of two TM proteins, MEL_183 and MEL_249. All inter-protein cross-links from structured regions comply with the 35 Å threshold. **E** Predicted structures of histone-like proteins MEL_368 (H4-H3 analog) and MEL_149 (H2B-H2A analog) as heterodimers and heterotetramers (stoichiometry inferred from prior knowledge of melbournevirus nucleosome data). Agreement of inter-protein cross-links and AlphaFold confidence score support the heterotetrameric model. All residue-specific cross-links are shown using XiNet^77^. Source Data are provided.

Having established the global melbournevirus interactome with sub-virion resolution, we next pursued atomistic structural insights. We predicted structures for all viral proteins in our cross-linking network (Fig. 3A) using AlphaFold3, focusing on proteins containing at least one intra-link (cross-links connecting lysines within the same protein sequence). Model quality was assessed using predicted template modeling (pTM) scores^5^.

To validate these predictions, we mapped intra-protein cross-links onto the AlphaFold3 structures and measured Cα-Cα distances (Supplementary Data 4). Models with low pTM scores (e.g., MEL_232, pTM = 0.1) frequently violated the DSSO distance constraint of 35 Å^47^, while high-scoring models (e.g., MEL_085, pTM = 0.95) showed excellent agreement with cross-linking data (Fig. 3B). Models with a pTM score of 0.5 and higher generally agreed with the cross-linking data and can therefore be considered high-confidence predictions. Overall, AlphaFold3 generated 45 high-confidence structures where >50% of intra-links satisfied distance restraints (Supplementary Data 4).

In addition to structures of individual proteins, we expanded our analysis to the prediction of heterodimers with AlphaFold3^5^. As a quality metric for the multimeric predictions, we used the model confidence score that combines pTM and ipTM scores. After filtering by this score, we identified 16 high-confidence dimeric models consistent with our cross-linking data (confidence score >0.5, at least 50% of inter-protein cross-links from structured regions satisfied, Fig. 3C and Supplementary Data 4). A notable example is the MEL_183:MEL_249 complex (confidence score = 0.72), where all seven inter-protein cross-links satisfied distance constraints (Fig. 3D). This model is further supported by the observation that both proteins are membrane incorporated (see also Fig. 2) and the globular domains building the interaction interface are both located in the coat.

Another insightful prediction is on the viral histone-like proteins. We obtained high-scoring heterodimeric models for MEL_368 (also known as the melbournevirus histone H4-H3-analog) with either MEL_369 (also known as the melbournevirus histone H2B-H2A-analog) or MEL_149 (also known as mini H2B-H2A-analog). While MEL_368:MEL_369 forms canonical nucleosome-like complexes within the virion, MEL_368 and MEL_149 association remained enigmatic in the viral context^14^ and were recently observed to form a heterotetrameric complex in vitro^17^. Comparative analysis of heterodimer versus heterotetramer predictions revealed a slight improvement of the confidence scores (from 0.78 to 0.86) and slightly increased cross-link concordance (from 4/6 to 5/6) (Fig. 3E). Compared to the heterotetrameric prediction of the canonical nucleosome complex MEL_368:MEL_369, the MEL_368:MEL_149 nucleosome is smaller, has fewer disordered loops, and a higher AlphaFold3 confidence score (Supplementary Fig. 3). The MEL_368:MEL_149 heterotetramer satisfied 5 out of 6 inter-link distance constraints, whereas most inter-links in the MEL_368:MEL_369 nucleosome complex exceeded the maximum distance cut-off. The overlength cross-links might represent interactions between neighboring MEL_368:MEL_369 heterotetramers as a result of nucleosome stacking, which is not reflected for the smaller MEL_368:MEL_149 complex. Collectively, these data support MEL_368:MEL_149 may form an alternative, minor nucleosome-like species that is present within native virions.

### High-confidence protein models pinpoint the putative functions of uncharacterized viral proteins

**Fig. 4:**
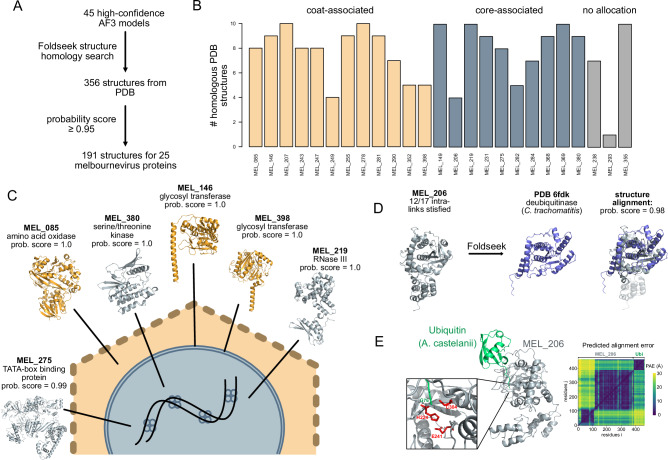
Structural comparison of high-confidence models with known PDB structures. **A** Pipeline for structure homology search of melbournevirus proteins. The AlphaFold models with high confidence scores (>0.5) and overall cross-link agreement (>half of intra-links satisfied) were used as templates for Foldseek analysis. After probability score filtering, 191 structures from the Protein Data Bank (PDB) were identified as structurally similar to 25 melbournevirus proteins. **B** Histogram of homologous PDB structures identified per melbournevirus proteins, colored according to sub-virion localization. **C** Structural similarity analysis using Foldseek, identifying matches between high-scoring melbournevirus protein models (probability score ≥0.95) and Protein Data Bank (PDB) entries. Selected hits are annotated with predicted molecular functions and virion localizations. **D** Representative structural alignment showing MEL_206 (gray) superimposed with a ubiquitin-specific protease from *Chlamydia trachomatis* (PDB: 6FDK^84^, blue). Right panel displays the alignment of both proteins with the corresponding probability score. **E** AlphaFold3 model of MEL_206 in complex with ubiquitin from *Acanthamoeba castelanii*. The orientation of ubiquitin to MEL_206 is in agreement with Ubi-DUB complexes, including the catalytic residues making contact with the ubiquitin C-terminal glycine (highlighted in red on the left). PAE plot (right) indicated medium to low confidence. Source Data are provided.

Leveraging our high-confidence structure models, we investigated potential functional roles of melbournevirus proteins through structural homology searches^48^. Using Foldseek^49^ we compared the 45 high-scoring, cross-link validated monomeric models against the Protein Data Bank (PDB) and AlphaFold databases. 25 models showed significant structural similarity (probability score >0.95) to known protein folds (Fig. 4A and Supplementary Data 5). With the exception of MEL_293, which only aligned with an uncharacterized marine metagenome sequence, all proteins showed similarity to multiple well-characterized PDB entries (Fig. 4B). Melbournevirus proteins located in the coat compartment generally share structural similarities with protein-modifying enzymes (10 out of 12 models allocated to the coat). Examples are amino acid oxidase (MEL_085) or glycosyltransferases (MEL_146 and MEL_398) (Fig. 4C). Proteins residing in the core, as expected, generally resemble proteins involved in nucleic acid-related processes (7 out of 10 models allocated to the core). For example, MEL_275 aligns with TATA-box binding proteins and MEL_219 has an RNase III-like structure.

The Foldseek predictions also hinted at yet undiscovered protein functions of individual proteins. For the core-associated putative serine/threonine protein kinase MEL_231, we found that, in addition to canonical kinase structures, its C-terminal region matches to an N-acetylglucosaminidase, suggesting a previously unrecognized dual functionality (Supplementary Data 5). Furthermore, coat-associated MEL_243, which currently lacks functional annotation, displayed strong structural similarity to fibronectin. We more closely examined core-associated MEL_206, another protein without prior functional annotation. The MEL_206 model aligned best with the structure of *Chlamydia trachomatis* DUB1 (deubiquitinase and deneddylase, Fig. 4D), suggesting potential ubiquitin protease activity. Supporting this hypothesis, the AlphaFold3 model of MEL_206 in complex with amoebal ubiquitin shows the typical active site residues of ubiquitin-specific proteases with a catalytic cysteine (C304) in the center of the predicted protease domain (Fig. 4E). These residues are proximal to the C-terminal glycine of ubiquitin, which facilitates the linkage to the target protein.

To query if MEL_206 may be involved in ubiquitination-related processes, we analyzed purified MelV particles by bottom-up proteomics and searched the data for peptides carrying a di-glycine modification, which signifies ubiquitination after tryptic digestion. We identified multiple di-glycine-modified peptides for the MEL_206 cross-linking partners MEL_368 and MEL_369 (Supplementary Data 6). These data suggest a possible importance for the ubiquitination status of viral proteins and their remodeling in the context of Melbournevirus infection. The data furthermore points towards MEL_206 being a strong candidate to play a role in ubiquitination remodeling and provide a starting point for future in vitro experiments to prove and study MEL_206’s potential deubiquitinase function.

### Refining the cryo-EM capsid structure by integrative modeling

In addition to putative enzymes, we sought to further characterize the structural properties of melbournevirus proteins. Current cryo-EM reconstructions of melbournevirus particles have resolutions ranging from 4.4 Å^26^ to 3.4 Å^50^. While certain regions of the map permitted de novo model building, the complexity of the system, unknown stoichiometry, and limited resolution in many other regions made de novo modeling based solely on density unfeasible^26,50^. To determine whether our integrative XL and structure modeling approach could facilitate structural analysis, we leveraged protein connectivity data from XL-MS to assign proteins in the 3.4 Å map^50^.

We began by fitting MEL_305 (MCP), the only previously assigned capsid protein^26^ into the map. Reassuringly, the predicted structure aligned well with the cryo-EM density, underscoring the accuracy of high-confident AlphaFold models (Supplementary Fig. 4).

**Fig. 5:**
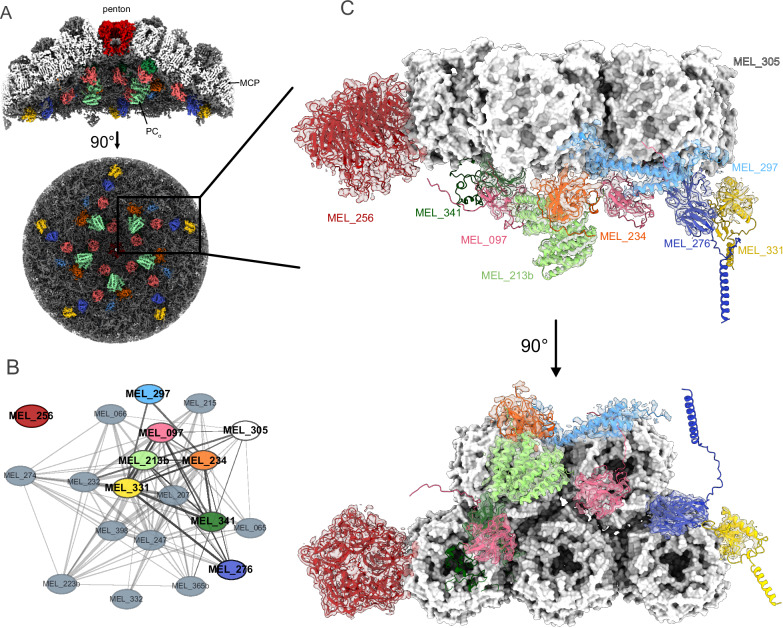
Structural annotation of the melbournevirus capsid using AlphaFold3 models and cryo-EM data. **A** The melbournevirus cryo-EM map focused on a five-fold symmetric capsid vertex (side and interior views, data from ref. ^50^). Different components of the capsid are color-coded in ChimeraX. **B** Part of the PPI network of the protein community located in the coat compartment and connected to MEL_305 or MEL_213b. Proteins MEL_256 and MEL_297 have also been added for completeness. Identified capsid-associated proteins are color-coded as in (**A**). Network was created using Cytoscape^76^. **C** Fitting of AlphaFold3 models into the cryo-EM density. Proteins are color coded the same as in (**A**) and (**B**). Major capsid protein MEL_305 is shown in surface-mode for clarity.

Beneath the MCP layer (Fig. 5A) is a supporting layer of minor capsid proteins forming a complex network. Among these, the pentasymmetron component (PCɑ) exhibits a rhodopsin-like stacked α-helical fold, though its identity remained unresolved in existing cryo-EM data^26^. To identify this protein, we screened high-scoring models among predicted coat proteins and found that density matched to MEL_213b, when predicted as a homodimer. Furthermore, homodimeric prediction of MEL_213b satisfied the XL-distance restraints (Supplementary Fig. 4).

To identify additional minor capsid components, we shortlisted candidate proteins from the coat community that cross-linked to either MCP or MEL_213b (Fig. 5B). By fitting these models into the cryo-EM map, we assigned MEL_234 and MEL_276 as minor capsid proteins (Fig. 5C). We also identified four other proteins - MEL_341, MEL_097, MEL_297 and MEL_331 - whose AlphaFold3 models contained well-structured domains consistent with the cryo-EM density. However, their lower accuracy domains could not be fitted, likely due to their high flexibility. In such cases, PPI data provided critical orthogonal evidence. Interestingly, MEL_276 and MEL_331 were predicted to be membrane-associated (see also Fig. 2C), suggesting a structural role in capsid-membrane tethering. Inter-protein cross-links of the fitted minor capsid proteins partially agreed with the proposed interfaces (Supplementary Fig. 5). The presence of over-length cross-links could either be due to protein flexibility or alternative assemblies of vertex complexes across the Melbournevirus capsid, which has been observed before for other giant viruses^29^.

Each vertex of the MCP icosahedron is characterized by the presence of a homopentameric complex, the penton^26^. Assigning the identity proved challenging, as none of the candidate models cross-linking to MCP or MEL_213b displayed a similar fold. We therefore screened all XL-MS detected proteins for potential homo-multimerization (*n* = 1–5, Supplementary Fig. 6 and Supplementary Data 7). As expected, MCP and MEL_213b scored the highest at their known stoichiometries. The only protein with a high-scoring pentameric model was MEL_256, which fit exceptionally well into the cryo-EM map (Fig. 5C). The detected inter-domain cross-link further supported the MEL_256 pentamer (Supplementary Fig. 4). Refinement of the AlphaFold3 models by tracing revealed an extensive contact between the penton complex and the minor capsid protein MEL_341, which wraps around the vertex center beneath the penton (Supplementary Fig. 7). Remaining unassigned densities (~21% of the volume of the original cryo-EM map) lacked sufficient evidence for confident model matching (Supplementary Fig. 8).

To extend these findings, we assessed the stoichiometry and symmetry of assigned capsid components using shotgun proteomics and intensity-based absolute quantification (iBAQ) values from purified virion samples. By calibrating copy numbers against four reference proteins (with known abundances from literature or our cryo-EM assignments), we obtained highly reproducible estimates across four technical replicates (Supplementary Fig. 9). This analysis suggested ~120 copies of MEL_097 and MEL_276, and ~ 60 copies of MEL_297 and MEL_341 (Supplementary Data 7). MEL_234 ( ~ 2200 calculated copies) and MEL_331 ( ~ 550 calculated copies) are incorporated in much higher stoichiometries and therefore are also likely part of the capsid outside the vertex, supporting the capsid faces or stabilizing contacts at the capsid edges (previously named scaffold component, glue or zipper^26,27^).

In summary, our integrative approach assigned seven minor capsid proteins as well as the penton protein using cross-link verified AlphaFold3 models. This demonstrated the power of combining XL-MS, structural prediction and proteomics to elucidate the structural details of the minor and major capsid proteins, their contact sites and membrane associations.

## Discussion

Since the discovery of the first giant virus two decades ago, many new NCLDV members have been identified, expanding our understanding of the boundaries of viral complexity^51–56^. With every newly discovered giant virus, more questions need to be addressed, urging the development of fast and systematic approaches to elucidate structure, proteomic arsenal, interaction with the hosts and ecological roles. To address this challenge, we developed an integrative approach combining XL-MS and AlphaFold3 modeling to comprehensively resolve the structural and spatial interactome of melbournevirus particles, providing unprecedented insights into its proteome organization.

Our XL-MS data partitioned the melbournevirus proteome into two spatially distinct communities separated by the inner membrane: an inner core and an outer coat (Fig. 1). While previous studies have provided insight into the overall protein content of giant viruses^23^, our spatially resolved interactome map delivers a more fine-grained picture of sub-virion composition. This information may help rationalize individual protein functions within either compartment. For example, MEL_196 resides within the core and interacts with histones and a putative helicase. Consistent with this spatial arrangement, the ortholog of MEL_196 in noumeavirus has been proposed to initiate and expand viral factories by undergoing liquid-liquid phase separation^57^.

We identified 25 TM proteins and annotated their topologies (Fig. 2). Through structural assignment on the cryo-EM density map, we assigned two TM proteins (MEL_331, MEL_276) as minor capsid proteins (Figs. 2 and 5). These proteins likely bridge the capsid and membrane through physical contacts, which may likely explain previous observations of membrane protrusions at the capsid vertices^27^. Notably, a similar membrane-capsid tethering architecture was observed in distantly related viruses like PBCV-1^28^ and medusavirus^58^, suggesting a conserved structural strategy.

While some viral enzymes from other giant viruses have been functionally validated^59–61^, the activities of individual melbournevirus-encoded proteins generally remain speculative and warrant in vitro characterization. Our results can provide enzyme activity predictions to guide such follow-up experiments. Using AlphaFold3, we generated high-confidence structural models (prioritized by pTM or confidence scores and cross-link validation) and assessed functional homology with Foldseek.

The coat community was enriched in proteins with predicted enzymatic functions, possibly acquired through horizontal gene transfer from the host^62^ or other viruses^63^. The presence of enzymes like proteases, isomerases, and glycosyltransferases in the coat community might indicate specific functions during capsid assembly. For example, it has been observed previously that giant virus capsids are glycosylated by virus-encoded glycosyltransferases^64^. Our analysis revealed that the coat proteins, MEL_146, MEL_290, and MEL_398 resemble glycosyltransferase folds and cross-link to minor capsid proteins MEL_213b, MEL_234, and MEL_331. This is consistent with the hypothesis that these two enzymes are involved in capsid protein glycosylation, most likely during capsid assembly.

The core community in contrast contains proteins resembling enzymes that might play a role in the replication (e.g., the predicted DNA glycosylase MEL_355) or interfere with host cellular mechanisms to promote viral replication (e.g., the RNase-III-like MEL_219). Interestingly, the model of core community protein MEL_206, which has no sequence homology and was completely uncharacterized prior to this study, shows structural homology to deubiquitinating enzymes. The core location of a potential deubiquitinase might point towards a protective role to prevent sensing of viral DNA, guarding viral core components and DNA-associated factors analogously to Herpes Simplex virus type 1 deubiquitinase UL36^65,66^. Overall, these observations suggest that core community proteins are primarily involved in the early infection when the genome and associated factors are released into the host cytoplasm.

Some predicted melbournevirus protein models, although having high confidence scores and high cross-link agreement, did not have any characterized homologous PDB structure (e.g., MEL_252, MEL_371, or MEL_293). In vitro characterization of these proteins in following studies might reveal novel folds and functions. Together, these examples highlight the utility of our approach for generating hypotheses to inspire follow-up investigations.

Beyond enzymes, we found a heterodimeric complex between MEL_149 and MEL_368 that forms an alternative nucleosome-like structure compared to the previously described MEL_368:MEL_369 complex^14^. Importantly, when MEL_149:MEL_368 was modeled as a heterotetramer, nearly all cross-links of the MEL_149:MEL_368 complex were satisfied, further validating the model. This prediction is consistent with a recently reported in vitro structure of MEL_149:MEL_368^17^. Our data further support the existence of this complex in intact virions. However, the MEL_149:MEL_368 complex likely exists in low stoichiometry, as MEL_149 is present at fewer than 100 copies per virion, which is much lower in comparison to the ~4,000 copies of MEL_368. Therefore, the MEL_149-containing complex may not significantly contribute to overall viral genome condensation, but it could play a regulatory role, potentially modulating transcription activation as observed in yeast^67^.

Finally, we demonstrated the power of our integrative structural modeling approach by assigning eight additional capsid components to a previously published cryo-EM map^50^. XL-MS data enabled us to generate a shortlist of potential protein candidates, significantly improving the success rate of matching models with the density. For regions with low-confident structural predictions or cryo-EM densities of limited resolution, the additional constraints provided by cross-linking data substantially increase assignment confidence.

We further improved our cryo-EM assignments using two complementary approaches: (1) protein copy number quantification through proteomics, and (2) homomultimeric state predictions using AlphaFold3^68^. These methods successfully confirmed known assemblies, including the trimeric MCP, homodimeric MEL_213b and homopentameric MEL_256. Additionally, we predicted homo-oligomerization for putative enzymes such as MEL_219 and MEL_168, both of which share structural similarity with known enzymes of defined homo-oligomeric states^69,70^.

While we were unable to match all remaining capsid densities to our predicted models—potentially due to protein flexibility or limitations in AlphaFold3 predictions—the PPI network and copy number analysis strongly implicate MEL_052, MEL_066, MEL_215, MEL_232 (vertex-associated), as well as MEL_223b and MEL_274 (at the capsid faces or edges) as capsid-associated proteins. These candidates represent prime targets for future structural characterization of the melbournevirus capsid.

In summary, we presented an integrative workflow combining XL-MS, proteomics, cryo-EM, and computational modeling to gain system-level insights into the spatial and structural interactome of the melbournevirus particles. Importantly, our approach only requires native virion particles, works without genetic manipulation, and is not limited by virus size or complexity. Therefore, it can be easily transferred to other *Marseilleviridae*, NCLDV members, and even micron-range giant viruses without major modifications of the protocol and pipeline. Likewise, other emerging pathogens outside the giant virus family are attractive targets for this powerful platform, enabling fast high-throughput characterization of understudied biological systems.

## Methods

### Propagation of melbournevirus in *Acanthamoeba castellanii*

Purified melbournevirus was produced as previously described^19,25^. In brief, *Acanthamoeba castelanii* (strain Neff, ATCC 30010) cells were cultured in PPYG medium (2.0% w/v proteose peptone, 0.1% w/v yeast extract, 4 mM MgSO4, 0.4 mM CaCl_2_, 0.05 mM Fe(NH4)2(SO4)2, 2.5 mM Na2HPO4, 2.5 mM KH2PO4, and 100 mM sucrose, pH 6.5) at 28 °C in a 5% CO_2_ incubator. Once the cells reached 100% confluency in ten 75 cm^2^ cell culture flasks, each flask was infected with 10 µL of seed melbournevirus. After three days of infection, the infected culture fluid (ICF) was harvested for purifying melbournevirus particles.

### Purification of melbournevirus particles for bottom-up and cross-linking proteomics

A total of 100 mL of infected cell culture fluid was centrifuged at 600 × *g*, 4 °C for 15 min. The supernatant was further centrifuged at 8500 × *g*, 4 °C for 35 min. The virus pellet was resuspended in 475 µL PBS, cross-linked with 5 mM freshly prepared DSSO in anhydrous DMSO, and incubated for 30 min at room temperature with shaking. Cross-linking was quenched with 15 µL of 1 M Tris (pH 8) for 30 min. The sample was rinsed once with 50 mL of 0.22-µm membrane-filtered PBS and centrifuged at 8500 × *g*, 4 °C for 35 min. The pellet was resuspended in 1 mL of PBS and applied to a 10–60% w/v sucrose gradient. Centrifugation was performed at 8500 × *g*, 4 °C for 90 min using an ultracentrifuge (Beckman Courter, Optima XPN-100, Sw40Ti rotor). The virus band was collected and rinsed three times with 50 mL of PBS by pelleting between rinses. Finally, the pellet was thoroughly resuspended in 100 µL of lysis buffer containing 8 M Urea, 1% v/v Triton X-100, 5 mM TCEP, 50 mM TEAB (pH 8). The sample was stored at −80 °C until further processing.

### Sample preparation for bottom-up proteomics

Lysis of melbournevirus particles was facilitated by sonication in a Bioruptor pico system at 10 °C using 30 s pulses of sonication, followed by 30 s of pause in 10 repetitions. Genomic DNA was digested by addition of 1 µl Benzonase per sample, followed by incubation for 30 min at room temperature. Afterwards, proteins were extracted and precipitated by methanol/chloroform precipitation. Protein pellets were resuspended in a buffer containing 1% (w/v) SDC and 5 mM TCEP as well as 40 mM Iodoacetamide in TEAB. Trypsin and Lys-C were added in a protein to enzyme ratio of 1:25 and 1:100, respectively, and digestion was carried out overnight at 37 °C while shaking at 1000 rpm. Next, digestion was stopped by acidification of the sample with 1% (v/v) formic acid. After centrifugation, peptides were desalted using C18 stage-tips^71^ and samples were dried by speed vacuum and stored at −20 °C until LC-MS measurement.

### Sample preparation for XL-MS

Sample preparation of cross-linked viral particles was carried out analogously to the bottom-up proteomics samples, with the exception that samples were sonicated with a Sonopuls HD 2070 Sonicator equipped with a MS73 Sonotrode (Bandelin) on 50% intensity for 30 s switching between sonication and pause every 0.5 s. After digestion, samples were desalted using c8 Sep-pak cartridges and peptide amount was determined by Pierce quantitative colorimetric assay (Thermo Fisher Scientific). After drying in speed vacuum, approximately 300 to 500 μg of peptides were loaded onto a PolySULFOETHYL A column (PolyLC) connected to an Agilent 1260 Infinity II system and peptides were separated using a 95 min gradient from buffer A (0.1% TFA in 20% ACN) to buffer B (0.5 M NaCL and 0.1% TFA and 20% ACN) while collecting fractions in 60 s intervals. All fractions were then desalted individually by stage-tipping, dried in speed vacuum and stored at −20 °C before LC-MS measurement.

### LC-MS measurement

Bottom-up proteomics samples were measured on an Orbitrap Fusion Tribrid instrument operating with Tune 4.0 and Xcalibur 4.6 and online-connected to an Ultimate 3000 RSLC nano LC system (Thermo Fisher Scientific). Peptides were run over an in-house packed c18 column (column material: Poroshell 120 EC-C18, 2.7 µm; Agilent Technologies) with a flow rate of 250 nl/min using a 180 min gradient going from buffer A (0.1% FA in water) to buffer B (0.1% FA in 80% Acetonitrile). Instrument parameters were set as follows: MS1 Orbitrap resolution 120,000, Scan-Range 375–1500 m/z, AGC-target “standard”, maximum injection time 50 ms. Duty cycles were set to 1 s. Intensity threshold for precursors was set to 1.0E + 4. Precursors of Charge 2–4 were selected with dynamic exclusion of 40 s and fragmented by Higher collisional energy dissociation (HCD) with normalized collision energy set to 30%. MS2 scans were recorded in the Ion Trap with scan rate set to “rapid” and scan range set to “auto”. AGC-target for MS2 was set to “standard”.

Cross-linking proteomics samples were measured on an Orbitrap Fusion Lumos mass spectrometer (Thermo Fisher Scientific) equipped with a FAIMS Pro Duo interface (Thermo Fisher Scientific) operating with Tune 4.0 and Xcalibur 4.6. Separation was performed on an in-house packed C18 column using the same LC gradient and flow as for bottom-up samples. FAIMS compensation voltages were set to −50, −60, and −75 V. Instrument parameters were set as follows: MS1 Orbitrap resolution 120,000, Scan-Range 375-1600 m/z, AGC-target “standard”, maximum injection time 50 ms. Duty cycles were set to 2 s. Intensity threshold for precursors was set to 2.0E + 4. Charge state filter for precursor selection was set to 4–8 and dynamic exclusion set to 60 s. Precursors were fragmented by stepped-HCD with normalized collision energy set to 21%, 27%, and 33%. MS2 scans were recorded in the Orbitrap with resolution set to 60,000 and scan range set to “auto”. AGC-target for MS2 was set to 200% and maximum injection time was set to 118 ms.

### Bottom-up proteomics data analysis

Raw files were searched with MaxQuant version 2.6.7.0^72^ with standard parameters, match between runs and iBAQ enabled. Methionine oxidation and N-terminus acetylation were set as variable modifications, carbamidomethylation of cysteine was set as fixed modification. Trypsin was selected as a digestion enzyme. Precursor mass tolerance was set to 20 ppm and fragment mass tolerance was set to 0.5 Da. Spectra were searched against the melbournevirus proteome database (TaxonID: 1560514^19^) as well as a database of the host *Acanthamoeba castellanii* previously described to be assembled from the reference database plus sequences received by RNA sequencing^40^. Results were filtered to 1% FDR at PSM and protein level.

For targeted search for protein ubiquitination, the bottom-up proteomics raw files were analyzed with Fragpipe (v22.0) MSFragger using the standard ubiquitin modification workflow (K, +114.04293 Da)^73,74^.

### Copy number estimation of virion-incorporated proteins

Calculation of copy numbers was conducted in R as described before^36^. Protein iBAQ-values from the proteinGroups.txt file were used for quantification. Proteins MEL_305, MEL_236, MEL_213b, and MEL_256 were used as internal standards with known copy numbers obtained from previous findings of cryo-EM (MEL_305: 9240 copies, MEL_236: 9240 copies, MEL_213b: 120 copies, MEL_256: 60 copies). For each of the 4 technical replicates a linear regression of log10 iBAQ values against log10 absolute copy numbers per virion was calculated. Copy numbers of remaining proteins were calculated using the slope and offset of the individual regression grade according to Eq. (1):

1 $${copy}\,{number}\,=\,1{0}^{(log 10({iBAQ})-{offset})/{slope}}$$

The mean of the calculated copy numbers from the 4 replicates was used.

### XL-MS data analysis

Raw files of XL-MS samples were searched using Scout software (version 1.5.1)^75^ against a database assembled from all melbournevirus proteins and all host proteins identified in the bottom-up proteomics experiments. The following parameters were selected: MS1 tolerance 10 ppm, MS2 tolerance 20 ppm, minimum peptide length 6 amino acids, maximum peptide length 60 amino acids, 3 missed cleavages and 2 variable modifications. Cross-linker type pre-settings for DSSO were used (158.00376533 Da, short arm 54.01056468 Da, long arm 85.98263585 Da) with residues K, S, T, Y and protein N-terminus as possible cross-linking sites. Results were reported at a 1% FDR at residue-pair-level and then aggregated to PPIs. Network representation of PPIs were made in Cytoscape^76^ and residue-specific representation of cross-links were created in XiNet^77^.

### Transmembrane domain prediction

All transmembrane domain prediction software was installed and run locally on Ubuntu 22.04.1 LTS using the melbournevirus protein sequences from the Uniprot database as input files. DeepTMHMM was run using pybiolib package with standard workflow described at https://dtu.biolib.com/DeepTMHMM. Probability files were used as results. DeepTMpred was run with python using model files supplied at https://github.com/ISYSLAB-HUST/DeepTMpred. Probabilities were extracted from JSON result files. TMbed was run using python with standard parameters as described at https://github.com/BernhoferM/TMbed. Results were generated in output-format 3 and probabilities were extracted from .pred files.

### Structure prediction with AlphaFold

AlphaFold version 3.0.1^5^ was installed and run locally on Ubuntu 24.04.1 LTS by utilizing the Docker platform. Predictions were run on a NVIDIA RTX A6000 GPU (48 GB RAM). Number of recycles was set to default. We used the initial AlphaFold3 input format, the default parameters which included the structural templates based on the RCSB Protein Data Bank as well as databases supplied at https://github.com/google-deepmind/alphafold3. Model parameters were received directly from Google. Number of multimer predictions per model was set to 1. The heteromultimeric model of MEL_149 and MEL_368 has been predicted with the AlphaFold3 online search server. pTM and ipTM scores were extracted from .json files using R and PAE data was extracted using python 3. For multimeric prediction the AlphaFold3 composite model confidence score^78^ was assessed as defined according to Eq. (2):

2 $${model}\,{confidence}\,=\,0.2\, * \,{pTM}\,+\,0.8 * \,{ipTM}$$

For every monomer or multimer prediction the model with the best agreement with cross-linking data was selected. In case of equally satisfied cross-links, the model with highest pTM, or in case of multimeric predictions, model confidence score was selected for analysis. Cross-links were plotted on predicted structures in R using the Bio3D package^79^ and visualized in PyMol (Schrödinger LLC, version 2.6).

Search for structurally related proteins was conducted using Foldseek^49^. This algorithm returns characterized protein structures from PDB and also the AlphaFold database that share structural similarity to the input model. The quality of the alignment was addressed on the basis of the given scores (e.g., TM-score or probability score).

### Interpretation of cryo-EM data

For comparison of predicted models with cryo-EM data the 3.4 Å resolution map of the melbournevirus capsid vertex described in Burton-Smith et al.^50^ has been loaded in UCSF ChimeraX v1.8^80^. Pictures were taken at volume step 1 and threshold level 0.012. Predicted AlphaFold3 models were loaded and fitted into the map using the “fit in map” tool. For predicted models with lower-confidence parts only the well-predicted parts were used for the fitting. Zone maps of the EM map around the fitted models are displayed as a transparent surface in a radius of 3 Å around the protein models.

For refinement of AlphaFold models fitted into the global capsid density map, the corresponding local area of the map was masked and saved as a new volume. For each of these new maps, the target density within the mask region was shifted to the center of the box followed by resampling the map to a smaller box size. Each model was then rigid-body docked into the resampled map and refined in ISOLDE^81^ with distance and angular restraints enabled. Some stretches of the models were manually tacked into the density and rebuilt. Residues for which no matching density could be found were deleted. Adjusted models were then real-space refined against the corresponding maps in phenix^82^ with default parameters and secondary structure restraints enabled. For the MCP trimer and pentamer models’ symmetry constraints and refinement of symmetry operators were included as well.

### Reporting summary

Further information on research design is available in the Nature Portfolio Reporting Summary linked to this article.

## Supplementary information

Supplementary Information

Description of Additional Supplementary Files

Supplementary Data 1

Supplementary Data 2

Supplementary Data 3

Supplementary Data 4

Supplementary Data 5

Supplementary Data 6

Supplementary Data 7

Supplementary Data 8

Reporting Summary

Transparent Peer Review file

## Source data

Source Data

## Data Availability

All raw files corresponding to this manuscript have been uploaded and made public to the ProteomeXchange Consortium via the PRIDE^83^ partner repository with the dataset identifier PXD064933. AlphaFold3 models have been deposited at Figshare [10.6084/m9.figshare.29290655]. Source data are provided with this paper.
