# Supplementary material for: Integrative structural interactomics reveals protein organization and structure in a giant virus
Source: Nat Commun. 2026 Jul 13;17:6139. doi: 10.1038/s41467-026-74973-2 (PMC13365592; doi:10.1038/s41467-026-74973-2)
Supplement: Supplementary file 1 — Supplementary Information [file 41467_2026_74973_MOESM1_ESM.pdf]

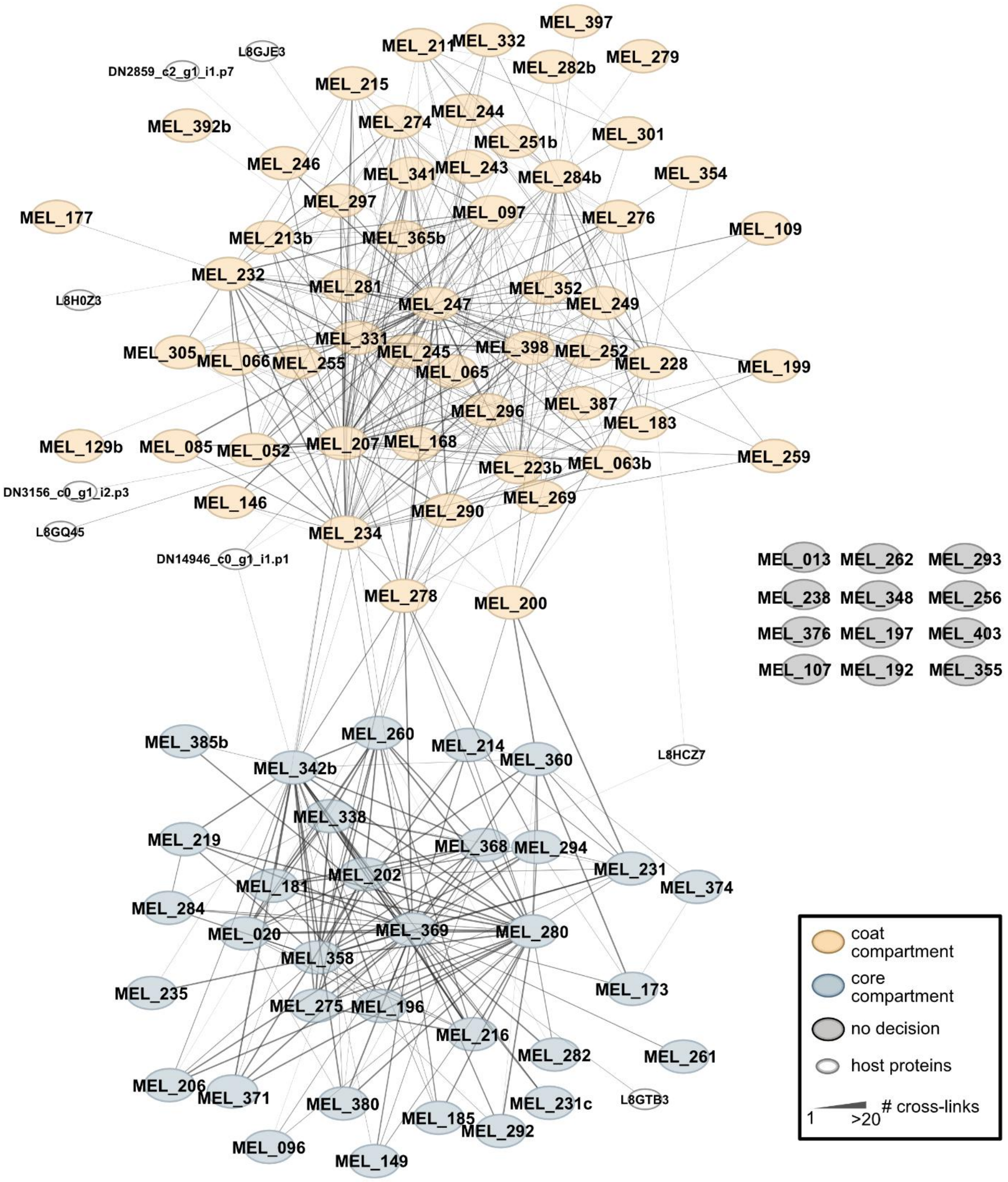

Supplementary Figure 1. Extended view of the melbournevirus protein interactome

XL-MS-based interaction network of melbournevirus as presented in Figure 1C. Protein gene names are annotated corresponding to the gene names from the Uniprot database. For host proteins lacking assigned gene names, Uniprot identifiers or transcriptomics-derived database annotations were used instead. Network was created using Cytoscape [76]. Source Data are provided.

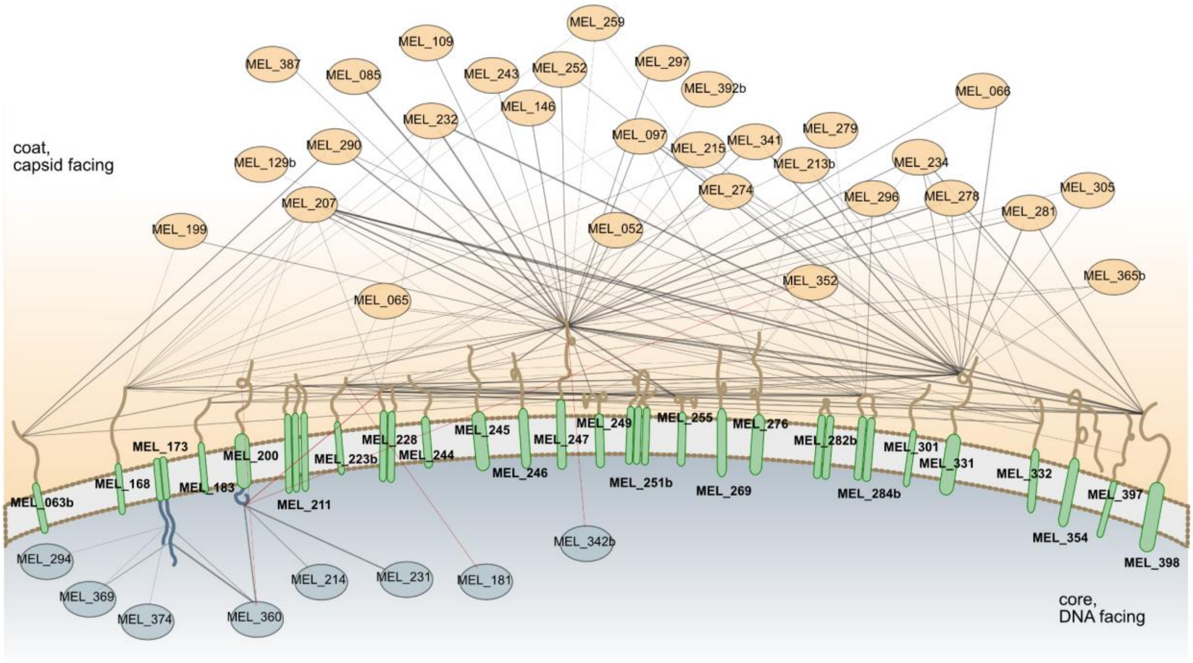

Supplementary Figure 2. Extended view of the membrane-proximal protein interactome of predicted membrane proteins

Overview of predicted transmembrane proteins, their topologies and their direct interaction partners, with all gene names annotated. Proteins are color-coded as in Figure 2B and 2C. For clarity, cross-links within the same domain targeting the same interactor are grouped. Network was created using Cytoscape [76]. Source Data are provided.

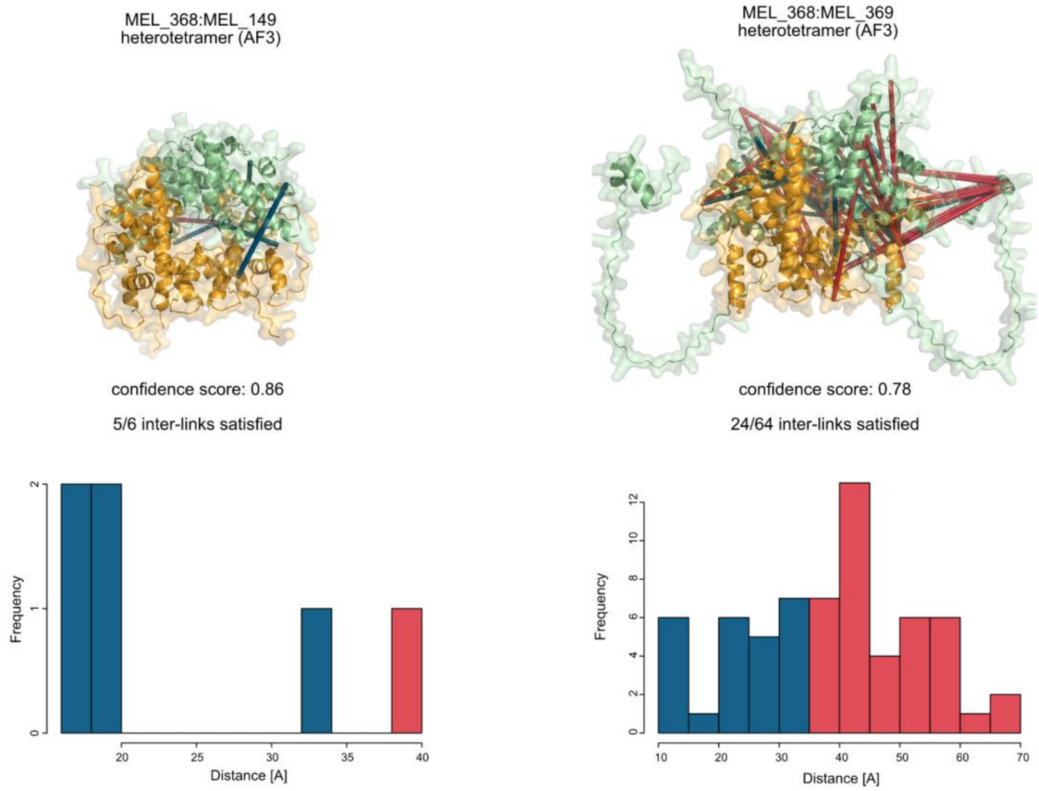

Supplementary Figure 3. Comparison of AlphaFold3 heterotetramer models of the smaller MEL_368:MEL_149 nucleosome-like complex and the canonical MEL_368:MEL_369 complex.

MEL_368:MEL_149 (left) complex is more compact and predicted with higher confidence compared to the MEL_368:MEL_369 complex (right). Inter-links plotted on the structures are colored according to meeting the DSSO distance criteria of 35 Å (blue satisfied, red violated). Source Data are provided.

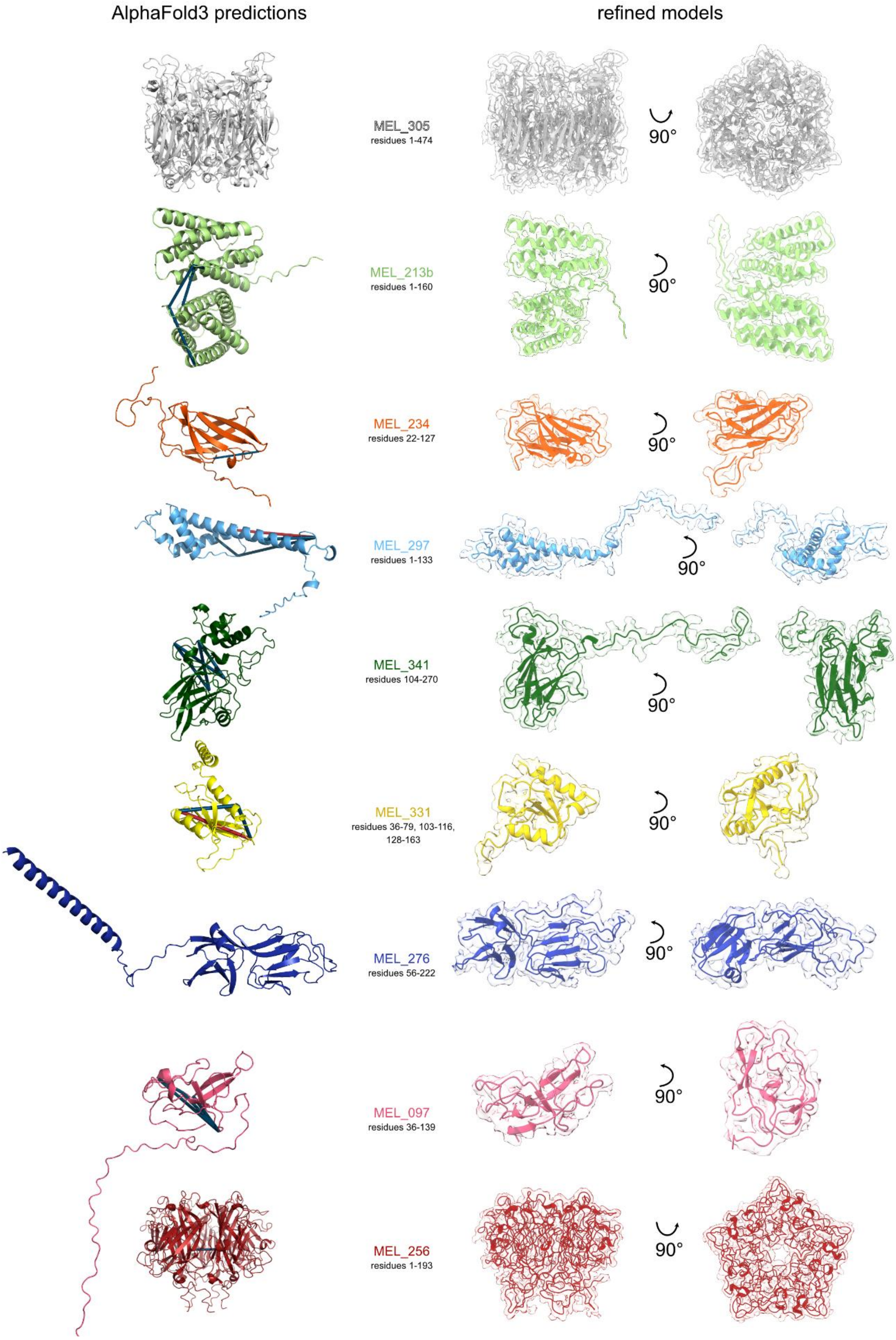

Supplementary Figure 4. Fitting refined AlphaFold3 models into cryo-EM density
AlphaFold3 models of the MCP (MEL_305), minor capsid proteins and penton protein in their assigned stoichiometry in this study, with mapped intra-protein cross-links

(left). Cross-links satisfying the DSSO distance constraint (≤35 Å, Cα–Cα) are shown in blue; over-length cross-links are in red. MEL_305 has no intra-link detected. MEL_276 only has intra-links from disordered regions. Cryo-EM density-guided refinement of models (right). Regions that could be traced are indicated below each protein name. Zone maps of the 5-fold symmetric Cryo-EM map are displayed as a transparent surface with a radius of 3 Å around the fitted protein structures. Source Data are provided.

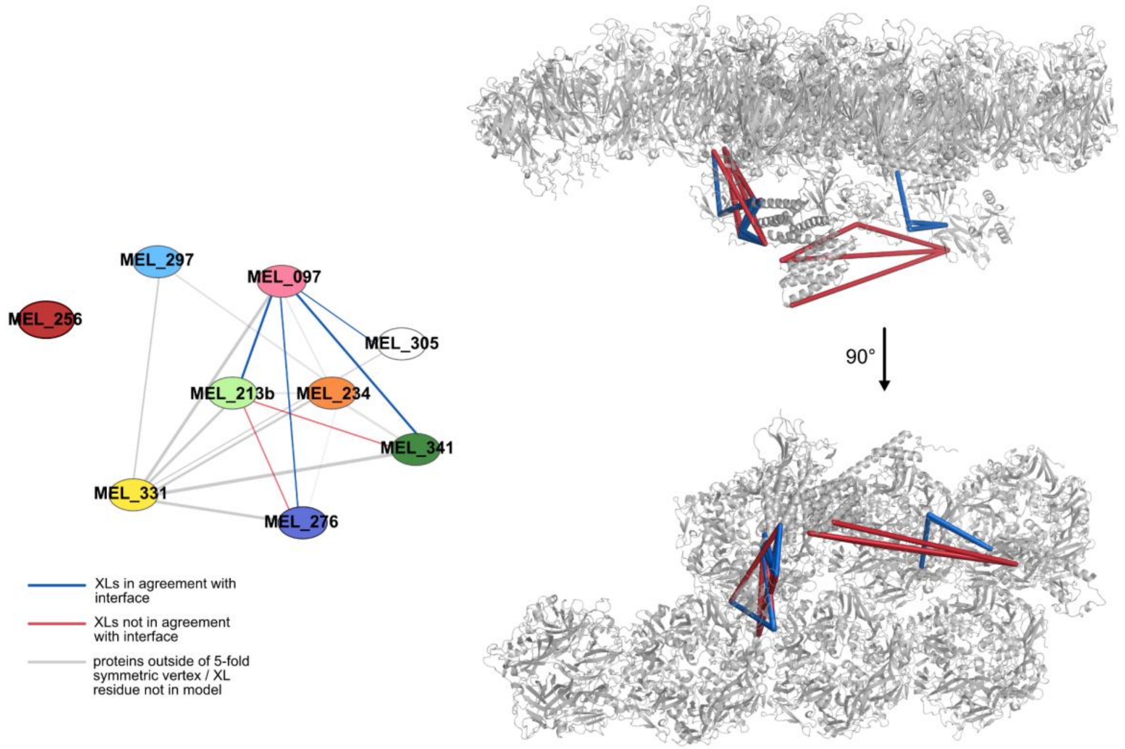

Supplementary Figure 5. Inter-inks of fitted minor capsid proteins partially agree with the proposed interfaces. AlphaFold3 models of the MCP, minor capsid proteins and penton protein are displayed in grey in the same conformation as displayed in Figure 5. Mapped inter-protein cross-links are colored according to the DSSO distance constraint. Only inter-links from minor capsid proteins specific for the 5 fold symmetric vertex are displayed. Proteins MEL_331 and MEL_234 are omitted from this analysis, because they are present in high stoichiometry (see Supplementary table 8) and possibly in multiple conformations. Network representation on the left summarizes which interfaces are in line with the detected cross-links and for which the majority of detected links is violating the DSSO distance criteria. Network was created using Cytoscape [76]. Source Data are provided.

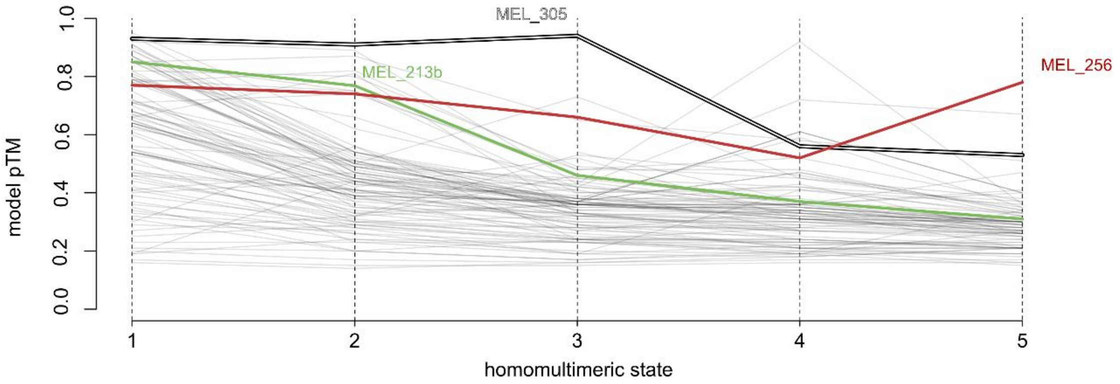

Supplementary Figure 6. Prediction of protein multimeric states using AlphaFold3 pTM scores.

Homomultimeric states were predicted for all proteins identified in the XL-MS dataset. The local maximum of the pTM score was used to determine the most likely oligomeric state. Two proteins - MEL_305 (MCP) and MEL_213b - with previously known stoichiometry as well as MEL_256 - the hereby identified penton protein - are highlighted. Source Data are provided.

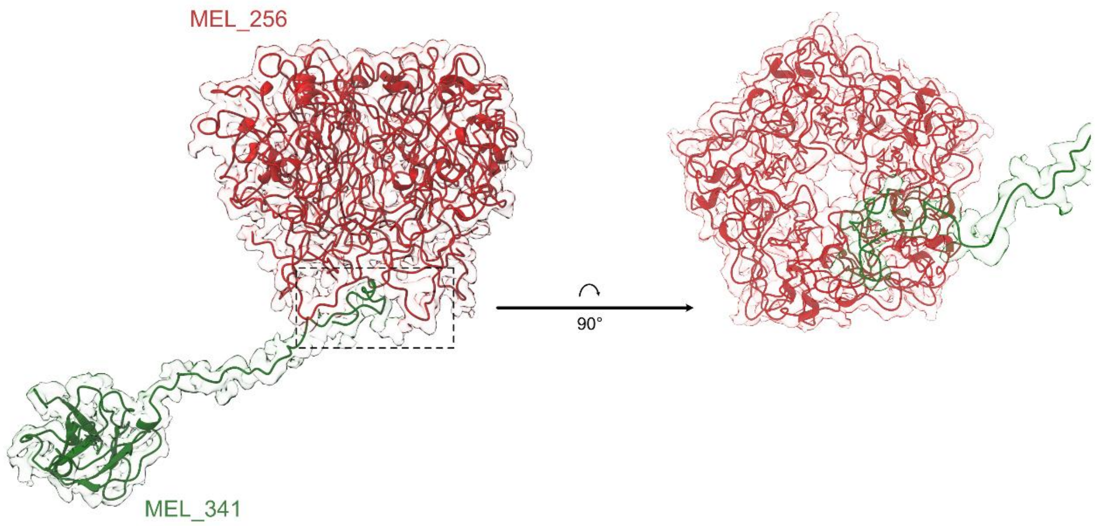

Supplementary Figure 7. Cryo-EM-guided structural refinement reveals interaction between MEL_341 and the penton protein

Refined AlphaFold models of pentameric MEL_256 (red) and MEL_341 (green) fitted into the cryo-EM density. A flexible region of MEL_341 (highlighted) mediates direct contact with the penton protein beneath the capsid vertex. Zone maps of the 5-fold symmetric Cryo-EM map are displayed as a transparent surface with a radius of 3 Å around the fitted protein structures.

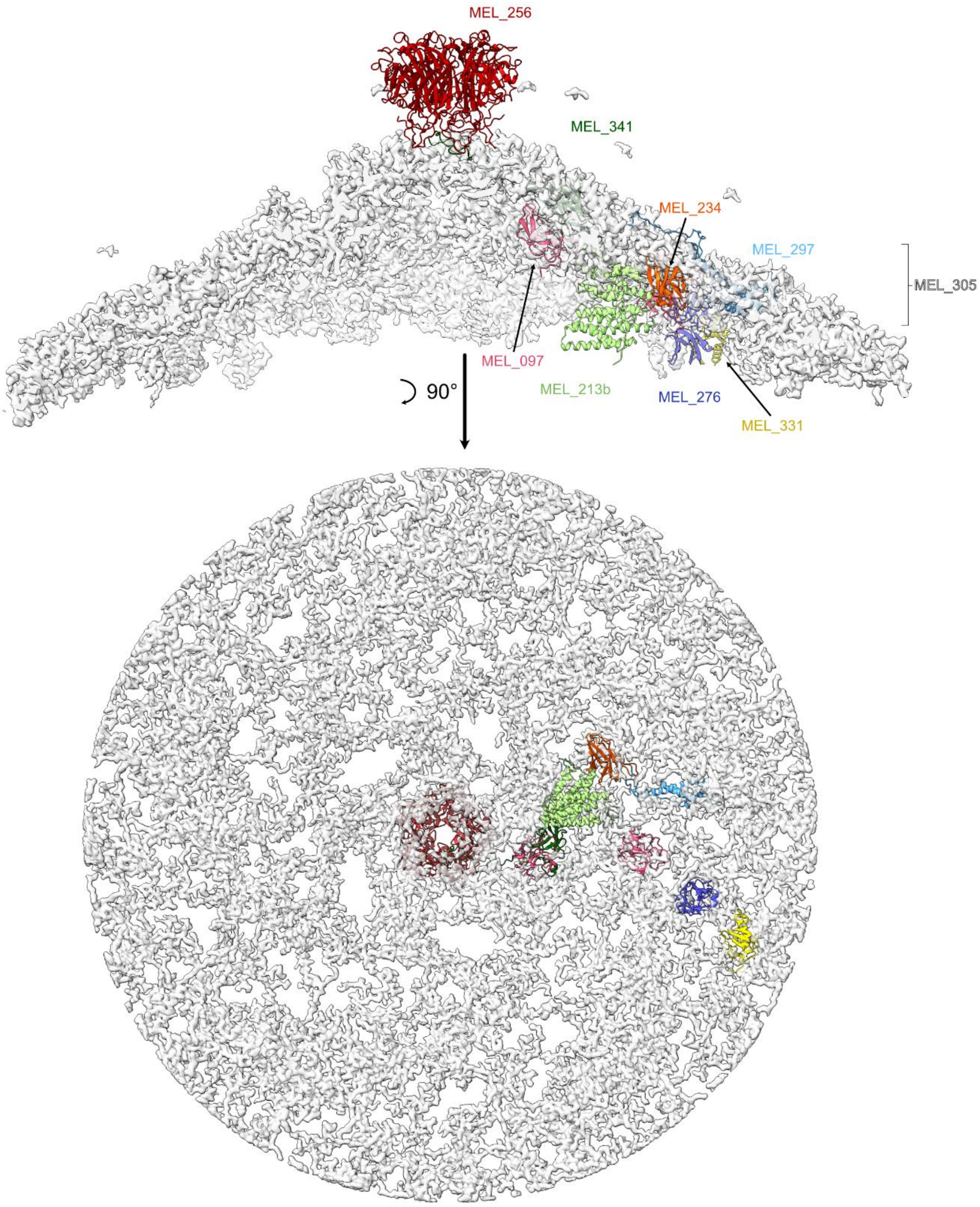

Supplementary Figure 8. Unassigned density in melbournevirus capsid vertex
Five-fold symmetric capsid vertex shown in side and interior views. The position of previously identified and newly assigned proteins are highlighted in different colors. Density around these identified components is removed. The remaining, yet unassigned density is shown in gray and corresponds to ~21 % of the complete density displayed in Figure 5.

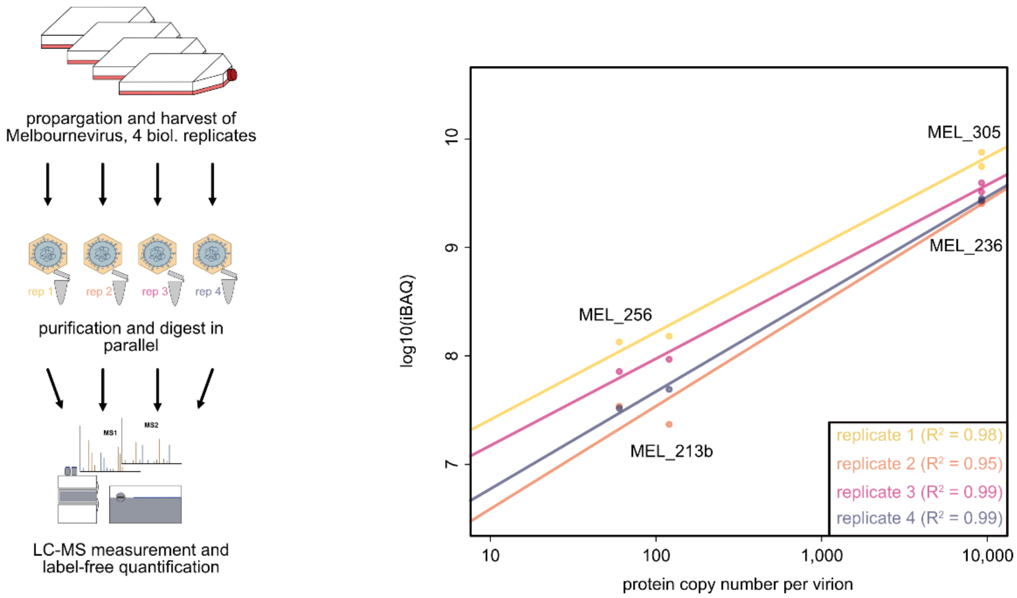

Supplementary Figure 9. Quantitative proteomics-based estimation of protein copy numbers

Protein iBAQ values were determined by label-free bottom-up proteomics across four replicates. Theoretical copy numbers were derived by plotting known capsid protein stoichiometries on a double-logarithmic scale. The linear relationship (reference lines shown) enables extrapolation of copy numbers for all other viral proteins based on their iBAQ values. Source Data are provided.
