# Supplementary material for: Integrative structural interactomics reveals protein organization and structure in a giant virus
Source: Nat Commun. 2026 Jul 13;17:6139. doi: 10.1038/s41467-026-74973-2 (PMC13365592; doi:10.1038/s41467-026-74973-2)
Supplement: Supplementary file 2 — Description of Additional Supplementary Files [file 41467_2026_74973_MOESM2_ESM.pdf]

Description of Additional Supplementary Files

File Name: Supplementary Data 1
Description: Table of detected cross-links per replicate and also of the combined, final dataset, as well as resulting protein-protein interactions (PPIs) and additional annotations of cross-linked viral proteins.

File Name: Supplementary Data 2
Description: Annotation of cross-linked proteins to core and coat compartment based on PPI network clustering.

File Name: Supplementary Data 3
Description: Annotation of transmembrane domains of cross-linked predicted TM proteins in the viral membrane with individual results from different TM domain prediction software.

File Name: Supplementary Data 4
Description: Agreement of intra-link data with monomeric AlphaFold predictions, as well as inter-link agreement of heteromeric AlphaFold predictions.

File Name: Supplementary Data 5
Description: Output of Foldseek structure similarity search of well-predicted models of viral proteins.

File Name: Supplementary Data 6
Description: Identified di-glycine modified peptides (ubiquitin signature modification) in viral samples.

File Name: Supplementary Data 7
Description: pTM scores of monomeric or homomultimeric (up to pentameric) AlphaFold predictions of Melbournevirus proteins from the PPI map to predict oligomeric states of viral proteins.

File Name: Supplementary Data 8

Description: Calculated per-virion copy numbers of incorporated proteins based on their iBAQ values using known copy numbers of capsid proteins as an internal standard. Theoretical and calculated copy numbers of identified capsid components is given.
