# Supplementary material for: Integrative structural interactomics reveals protein organization and structure in a giant virus
Source: Nat Commun. 2026 Jul 13;17:6139. doi: 10.1038/s41467-026-74973-2 (PMC13365592; doi:10.1038/s41467-026-74973-2)
Supplement: Supplementary file 12 — Transparent Peer Review file [file 41467_2026_74973_MOESM12_ESM.pdf]

nature portfolio

Peer Review File

Integrative structural interactomics reveals protein organization and structure in a giant virus

Corresponding Author: Professor Fan Liu

This file contains all reviewer reports in order by version, followed by all author rebuttals in order by version.

Version 0:

Reviewer comments:

Reviewer #1

(Remarks to the Author)
Review for Nature Communications NCOMMS-25-52784-T

The manuscript "Integrative structural interactomics reveals protein organization and structure in a giant virus" by Muhlberg et al describes crosslinking and mass spectrometry approach to understand protein:protein interactions within Melbournevirus, a member of the giant virus family Marseilleviridae. The authors identify a number of proteins as either associated with the capsid ("coat associated"), or within the inner membrane bound nucleocapsid ("core associated"). The authors then use a combination of alpha fold and combine the predicted structures into a previously deposited cryo-EM map, filling in knowledge gaps regarding the capsid organization. This is an especially important topic as many giant virus proteins are not well understood, or have known functions. In general this is a very powerful technique that can be widely applied to a variety of giants. That being said, I have a number of comments and suggestions that would strengthen the current work, and need to be addressed prior to publication. The work feels like a summary of what was done or a methods paper, with little links to the biology and significance of the study. It's a missed opportunity as this could be more impactful to the giant virus community with better discussion of the results.

The introduction is not very accessible to a broad audience. I would spend more text describing why Marseilleviridae and Melbourne virus in particular are significant to study. Given the breadth of giant viruses I would also point out the unique features within this giant virus compared to more well known giants.

The biggest concern I have is that the authors interpret data that is repeated in only 2 out of 3 replicates (~64 % of interactions) instead of taking the more conservative view of crosslinks that occur in all 3 replicates (50% of interactions). Since chemical crosslinking can be rather non-specific, there could be a significant amount of error—especially considering the yellow replicate in figure 1 that has a whopping 243 outliers. At the very least, the authors should explicitly state which proteins were only in 2 of 3 replicates, so that the readers would know which connections might be false positives, or more transient and not robust protein:protein interactions. This would greatly enhance the rigor of the work.

More detail is needed in several sections. For each protein listed, instead of a name that makes no sense to the general public (ex: MEL_206) I suggest to link the genbank accession number and some description of what was known prior to the identification in the crosslink in the main text. The data in the supplemental is fine, but very cumbersome to the reader to look up each one individually.

Also would be helpful to have some metric/discussion on how many new proteins were identified from the current work. Were they annotated before? Any conflicts with former hypothesis?
How much of the capsid is left as an unassigned volume now that several proteins are fitted into the density map? This is especially hard to understand with the current figures as there is so much symmetry. Similarly, does the mass spectrometry data correspond to the copy number of each protein that one could infer from fitting into the map? The stoichiometry can't be 1:1 with the major capsid protein for all the proteins. I suggest a table listing the copy number for each (or at least relative amounts).

Zone maps should be shown for each protein fitted into the capsid (2-3 alpha helices each) in order to determine how well the predictions line up with the data-can side chains easily be discerned for all? Without this information the quality of the

predictions is hard to assess.

I would also like to see some discussion on the scalability. Since Melbourne is one of the smaller giants (~250 nm capsid) how would this technique need to be modified for some of the larger virions (some can go several microns)? What about other features. Did fibril crosslinks occur as well? Can this inform about fiber attachment sites to the capsid?

Reviewer #2

(Remarks to the Author)
In this manuscript, the authors utilized cross-linking MS, quantitative proteomics, and computational biology to characterize the protein architecture of melbournevirus particles. Novel biological insights were generated, including sublayer localization of 88 viral proteins, topological assignment of 25 inner membrane proteins, as well as mapping of 8 proteins in the cryo-EM structure of the capsid. This MS-enabled structural biology study seems thorough and high quality, with consistent results among cross-linking MS, AlphaFold modeling, and viral localization. The scope of the study is also suitable for Nature Communications. The authors might consider including, but not warranted, validation experiments on protein localization or enzymatic activity assignments, will further strengthen the study. Additionally, this reviewer has some minor suggestions for authors to consider:

1. Authors included less reactive amino acids, such as S, T, and Y, in cross-linked peptide identification, which has the danger to inflate false discovery rate. It will help to include discussion on the prevalence of these less reactive residues in identified peptides.
2. AlphaFold doesn’t have a confidence score of 0-1.0 range. ipTM scores were discussed in the Method section for multimeric prediction. Based on AlphaFold, ipTM scores below 0.6 suggest failed predictions. It will help to clarify the specific confidence scores described in the results.
3. A total of 16 high confidence models seem low for 88 viral proteins with cross-linking PPIs for sublayer assignment. It will help to discuss possible reasons for such discrepancy, such as the quality of AlphaFold predicted model, or cross-links in unstructured regions etc.

Reviewer #3

(Remarks to the Author)
I co-reviewed this manuscript with one of the reviewers who provided the listed reports. This is part of the Nature Communications initiative to facilitate training in peer review and to provide appropriate recognition for Early Career Researchers who co-review manuscripts.

Reviewer #4

(Remarks to the Author)
This work demonstrates the utility of crosslinking mass spectrometry to rapidly provide structural information in poorly understood biological systems, in this case, the giant Melbournevirus. The study provides structural insights using an integrative approach that combines XL-MS with AlphaFold-based modeling to improve our understanding of the architecture of this understudied and complex virus. I am excited to see the use cases of these techniques expanding into different biological samples.
However, the impact of this study relies on its addition to the body of knowledge on Melbournevirus biology so the paper needs to be clearer on the structural implications of the discovered structural folds and protein-protein interactions. P

Major concerns:

The authors should include more discussion on the biological implications of the discoveries made in this study.

The authors use XL-MS as a type of spatial proteomics approach that will indicate which side of the membrane proteins reside on. Why do the authors think that some membrane proteins crosslink to so many proteins on the capsid-facing side of the membrane? Are these non-specific crosslinks?

The section on high-confidence AF3 models with Foldseek leaves the reader wanting much more detail. This section requires some validation of the structurally predicted function of these proteins. In particular, the proposed debiqutinase activity of MEL_206 should be assayed.

The proposed new histone-like viral protein pair MEL_149-MEL_368 is not discussed in enough detail. Does it retain the features of other nucleosome-like structures, such as charged patches for binding DNA?

Crosslinks have been very often used in the past to help refine model building into cryo-EM densities using crosslinks as distance restraints. The section on expanding the fitted model into the cryo-EM density is interesting, but I wonder if this could have been achieved with AF3 alone using a trial-and-error approach? The crosslinks as distance restraints are not used or shown at all. Do the crosslinks not agree with the suggested interfaces? Can the crosslinks be used to confirm interfaces that are shown and suggest where other proteins should be localised?

Minor comments:

Throughout the manuscript the authors need to be clearer about which metrics they are extracting from AF3 and which they consider to be high confidence. Throughout the manuscript, "confidence score" is used without being defined. Please check every usage throughout the manuscript and improve this in the Methods.

Line 202 “supplementary Tab 3” should be “supplementary Table 4"

Line 220 “Another insightful prediction is on the viral histone-like proteins. We obtained high-scoring heterodimeric models for MEL_368 (also known as the melbournevirus histone H4-H3-analog) with either MEL_369 (also known as the melbournevirus histone H2B-H2A-analog) or MEL_149 (also known as mini H2B-H2A-analog). While MEL_368:MEL_369 forms canonical nucleosome-like complexes within the virion, MEL_368 and MEL_149 association remained enigmatic 14 and were only recently observed to form a complex in vitro 42.”
The crosslinks and AlphaFold models for MEL_368:MEL_369 appear directly comparable to the MEL_368:MEL_149 analysis. Please include the MEL_368:MEL_369 crosslink mapping and model evaluation alongside MEL_368:MEL_149 (e.g., distance-satisfaction statistics, any conflicting links, and a brief structural comparison).

Line 281 “However, their lower accuracy domains could not be fitted, likely due to their high flexibility.”
The statement “their lower-accuracy domains could not be fitted, likely due to high flexibility” is reasonable, but you might further test biological context by running AlphaFold 3 with adjacent interactors included in the same input (i.e., co-modeling subsets of the complex). In many cases, AF-Multimer improves interface accuracy when co-evolutionary context is provided. If feasible, please report whether co-modeling improves the problematic domains or interface confidence.
Figure 5 For interpretability, please overlay inter-protein crosslinks directly on the structural model in Figure 5 (or add a supplementary panel). Distinguishing inter- vs intra-links visually (e.g., separate colors/line styles) would help readers connect the XL evidence to the proposed interfaces.

Line 298 “Remaining unassigned densities in the cryo-EM map lacked sufficient evidence for confident model matching”
Given the strength of your XL dataset, have you evaluated AlphaLink to help assign proteins to the remaining unmodeled cryo-EM densities?

Reviewer #5

(Remarks to the Author)
I co-reviewed this manuscript with one of the reviewers who provided the listed reports. This is part of the Nature Communications initiative to facilitate training in peer review and to provide appropriate recognition for Early Career Researchers who co-review manuscripts.

Version 1:

Reviewer comments:

Reviewer #1

(Remarks to the Author)
I am satisfied with the authors' responses to my critiques. Congrats on a lovely methods paper that will be important to the giant virus community.

Reviewer #3

(Remarks to the Author)
The authors have sufficiently addressed the major concerns that were identified in the initial review. Thus, we enthusiastically recommend this manuscript for publication. Some minor improvements that may strengthen the manuscript include:

1. Adding supplementary figures where both intra-links and inter-links are shown on the highlighted structures addressed in the main figures, including both violation and fit color schemes. (example intra-link violating distance cutoff light pink; intra-link fitting distance cutoff dark pink. Inter-link violating distance cutoff light green; inter-link fitting distance cutoff dark green.) This would utilize and present the valuable structural information that long-range sequence space intramolecular crosslinks hold.

2. Although it could be implied from the statement that the default AlphaFold settings were used, it would be more transparent to explicitly mention the template settings used (templates or template free) in the AlphaFold predictions in the methods section.

Reviewer #4

(Remarks to the Author)
The Authors have answered all of our concerns and the manuscript is now suitbale for publishing,

Open Access This Peer Review File is licensed under a Creative Commons Attribution 4.0 International License, which permits use, sharing, adaptation, distribution and reproduction in any medium or format, as long as you give appropriate credit to the original author(s) and the source, provide a link to the Creative Commons license, and indicate if changes were made.
In cases where reviewers are anonymous, credit should be given to 'Anonymous Referee' and the source.
The images or other third party material in this Peer Review File are included in the article's Creative Commons license, unless indicated otherwise in a credit line to the material. If material is not included in the article's Creative Commons license and your intended use is not permitted by statutory regulation or exceeds the permitted use, you will need to obtain permission directly from the copyright holder.
To view a copy of this license, visit https://creativecommons.org/licenses/by/4.0/

We thank all five reviewers for carefully reading and evaluating our manuscript! We were particularly pleased to read that we address an “important topic” with a “powerful technique” (reviewer 1), that our work “seems thorough and high quality” (reviewer 2) and that reviewer #4 was excited to see the “use cases of these techniques expanding into different biological systems”.

The reviewers also raised important points, including the request to provide more background on the interesting biology of MelV (reviewer #1,#4), to provide additional validation experiments (reviewer #2,reviewer#4) and additional analyses on the provided XL-MS/AlphaFold data (reviewer #1,reviewer#4).

We have now carefully addressed these comments which led to extensive remodelling of the introduction and discussion parts of the original manuscript. Additionally we revised Figure 4 extensively and include now supplementary Figures 3 and 5 to illustrate some of the findings in response to reviewer #4. We are pleased with the current manuscript and feel that our study is now ready for publication in *Nature Communications.*

In all answers below, we refer to text revisions by indicating their line numbers as [lines XXX–XXX] in the revised manuscript.

Reviewer #1 (Remarks to the Author):

Review for Nature Communications NCOMMS-25-52784-T

The manuscript “Integrative structural interactomics reveals protein organization and structure in a giant virus” by Muhlberg et al describes crosslinking and mass spectrometry approach to understand protein:protein interactions within Melbournevirus, a member of the giant virus family Marseilleviridae. The authors identify a number of proteins as either associated with the capsid (“coat associated”), or within the inner membrane bound nucleocapsid (“core associated”). The authors then use a combination of alpha fold and combine the predicted structures into a previously deposited cryo-EM map, filling in knowledge gaps regarding the capsid organization. This is an especially important topic as many giant virus proteins are not well understood, or have known functions. In general this is a very powerful technique that can be widely applied to a variety of giants.

We thank the reviewer for taking the time to carefully review our manuscript and are grateful they review our research as “important” and the approach presented as “powerful”.

That being said, I have a number of comments and suggestions that would strengthen the current work, and need to be addressed prior to publication. The work feels like a summary of what was done or a methods paper, with little links to the biology and significance of the study. It’s a missed opportunity as this could be more impactful to the giant virus community with better discussion of the results.

We also agree with the reviewer that a more detailed discussion for the biological relevance of the findings strengthens our manuscript. Therefore, we extended the discussion section at the following instances.

(i) We expanded the biological context of the finding that predicted enzymes such as glycosyltransferases are located to the coat and nucleic acid related enzymes locate to the core. [lines 391-423]

(ii) we emphasized the scalability to other giant viruses. [lines 459-465]

(iii) we discussed how data relate to latest research findings of initiation of Marseilleviridae replication compartments by viral proteins undergoing liquid-liquid phase separation. [lines 376-382]

The reviewer also points out that parts of the manuscript read like a methods paper. We share this sentiment, since this manuscript describes a novel approach for elucidating protein localization, complexation and possible function, as well as annotating cryoEM structures based on XLMS data and AlphaFold modeling, requiring only a proteome or translated genome as *a priori* input. It is our intention to position this paper at the intersection of method development and biological discovery, which is precisely the reason why we feel this work fits very well to the broad scope of Nature Communications.

The introduction is not very accessible to a broad audience. I would spend more text describing why Marseilleviridae and Melbourne virus in particular are significant to study. Given the breadth of giant viruses I would also point out the unique features within this giant virus compared to more well known giants.

We adjusted our introduction to meet the reviewer's suggestion. We now include background info on unique Marseilleviridae features including:

(i) genome condensation [lines 78-83]

(ii) viral factories [lines 88-91]

(iii) capsid architecture [lines 99-103]

The biggest concern I have is that the authors interpret data that is repeated in only 2 out of 3 replicates (~64 % of interactions) instead of taking the more conservative view of crosslinks that occur in all 3 replicates (50% of interactions). Since chemical crosslinking can be rather non-specific, there could be a significant amount of error—especially considering the yellow replicate in figure 1 that has a whopping 243 outliers. At the very least, the authors should explicitly state which proteins were only in 2 of 3 replicates, so that the readers would know which connections might be false positives, or more transient and not robust protein:protein interactions. This would greatly enhance the rigor of the work.

The final PPI dataset used in the manuscript did in fact only contain interactions found in three out of three replicates (instead of only two out of three), as it is displayed in Figure 1 B and Supplementary table 1. This was wrongly stated in the results part of the original

manuscript (lines 136-137). We apologize for this mistake and thank the reviewer for pointing this out. We corrected the sentence in the results part accordingly:

“To minimize false positives, we retained only PPIs observed in all three replicates.”

Additionally, we understand the reviewer pointing out this seemingly high inconsistency in the biological replicates. The overlap of the individual replicates in our study is in the same range or higher as overlap of biological replicates previously reported in XL-MS studies: (Wheat, et al. PNAS 2021 https://doi.org/10.1073/pnas.2023360118, Fasci et al. MCP 2018 https://doi.org/10.1074/mcp.RA118.000924, Weisbrod et al. JPR 2013 https://doi.org/10.1021/pr3011638, Götze et al. AnalChem 2019 https://doi.org/10.1021/acs.analchem.9b02372). A major reason for the generally lower overlap between replicates in XLMS compared to conventional proteomics is that most cross-links are detected near the sensitivity limit of modern LC-MS approaches. Since detection of cross-linked peptides is done in a data-dependent acquisition method, this leads to the inevitable fact that many cross-linked peptides identified in one sample will be missed in another sample.

That being said, we politely disagree with the statement that “chemical crosslinking can be rather non-specific”. We and others have shown multiple times that cross-links, even if identified only in a single replicate, agree extremely well with published high-resolution protein complex structures and show near-perfect compartment specificity when applied to intact biological systems (e.g., O’Reilly FJ et al., Mol Syst Biol 2023 https://doi.org/10.15252/msb.202311544, Zhu et al., Nat Commun 2024 https://doi.org/10.1038/s41467-024-47569-x), arguing against a substantial presence of non-specific contacts in the datasets. In the present manuscript, the sparsity of structural and spatial information on MelV proteins prohibits a large-scale comparison of our XLMS results to published data. As an alternative means of validation, we confirm the internal consistency of our XLMS results, showing that 98.6% of the cross-links can be consolidated into a coherent membrane protein topology prediction (Figure 2D). We would also like to point out that replicate #1 having identified 243 interactions missed in the other two is not necessarily a sign for error but results from the simple fact that we had more identifications in this experiment. This was probably due to the reason that we could derive more material from the sample and could therefore reach higher intensities, resulting in the detection of cross-linked peptides that fell below the sensitivity threshold in the other replicates. Still, to ensure high-confidence data, we exclude those identifications from the final dataset with our stringent filtering strategy.

More detail is needed in several sections. For each protein listed, instead of a name that makes no sense to the general public (ex: MEL_206) I suggest to link the genbank accession number and some description of what was known prior to the identification in the crosslink in the main text. The data in the supplemental is fine, but very cumbersome to the reader to look up each one individually.

While we understand the reviewers discontent with the nomenclature of Melbournevirus proteins, as the ORF names are not implying any function, we decided to stick to these names as it is exactly how they were deposited in uniprot. We oriented ourselves at

comparable, discovery-driven interactomics studies (O'Reilly et al., Mol Syst Biol 2023 https://doi.org/10.15252/msb.202311544, Choi et al PLOS Biol 2019 https://doi.org/10.1371/journal.pbio.3000475) that also refer to gene names for simplicity. Additionally, since we highlighted our findings on unannotated proteins (minor capsid proteins, MEL_206) and therefore there was no meaningful protein name available. To make our findings easier to navigate, we now included Supplementary Table1 sheet 5 "Protein_Annotations" that adds additional literature based annotations.

Also would be helpful to have some metric/discussion on how many new proteins were identified from the current work. Were they annotated before? Any conflicts with former hypothesis?
We have limited our analysis to the 403 ORFs previously predicted for MelV (Doutre et al. JVirol 2014 https://doi.org/10.1128/jvi.02414-14). Prior work had provided protein evidence for 141 (Liu et al. Cell 2021 https://doi.org/10.1016/j.cell.2021.06.032) and functional predictions for ~250 ORFs (NCBI nucleotide, Uniprot, Doutre et al. J Virol 2014 https://doi.org/10.1128/jvi.02414-14). In our LC-MS data, we identified 176 ORFs – including 135 ORFs with previous protein evidence – and found 88 ORFs to be involved in cross-links. The sub-virion location and interaction partners of all functionally annotated ORFs agreed with previous predictions. In addition, the XLMS data allowed us to make functional predictions for 3 ORFs that had not been annotated before plus 8 unannotated ORFS now shown to participate in building the minor capsid protein network.

How much of the capsid is left as an unassigned volume now that several proteins are fitted into the density map? This is especially hard to understand with the current figures as there is so much symmetry.

Judged by the volume size measured in ChimeraX, the volume left unassigned (Supplementary Figure 6) corresponds to ~ 21 % of the volume in the original cryoEM map (5-fold symmetric capsid vertex, Figure 5 A).

Similarly, does the mass spectrometry data correspond to the copy number of each protein that one could infer from fitting into the map? The stoichiometry can't be 1:1 with the major capsid protein for all the proteins. I suggest a table listing the copy number for each (or at least relative amounts).

The reviewer is right. The stoichiometry of the capsid proteins (identified and only speculated ones) is not 1:1 to the major capsid protein. In fact, we used the per-capsid-copy numbers of the major capsid protein, the penton protein and PC-alpha protein to determine exact copy numbers of all virion incorporated proteins, as described in lines 300-310 of the original manuscript. Importantly, this regression analysis was only possible because the copy numbers of these three proteins span several orders of magnitude and therefore allow interpolation of unknown protein amounts. To increase the accessibility of this finding, we included another supplementary data Table 8, sheet 2, which indicates the stoichiometry of the fitted capsid proteins.

Zone maps should be shown for each protein fitted into the capsid (2-3 alpha helices each) in order to determine how well the predictions line up with the data-can side chains easily be discerned for all? Without this information the quality of the predictions is hard to assess.

The original manuscript included zone maps from the 5-fold symmetric CryoEM map in Figure 5, and Supplementary Figures 3 and 5 as the transparent surface around the fitted models. This surface represents the zone map in a 3 Å radius around the fitted AlphaFold model. This was not clear from our previous methods section and we added this information accordingly in the methods part and in the legend of all Figures displaying the zone maps.

I would also like to see some discussion on the scalability. Since Melbourne is one of the smaller giants (~250 nm capsid) how would this technique need to be modified for some of the larger virions (some can go several microns)?

We thank the reviewer for raising this important point. This approach is fully scalable to other giant viruses as long as there are proteins with lysine residues to cross-link. Since DSSO cross-linking is regularly applied to whole cells, also larger viruses (> several µm) are amenable to this technology with regard to both cross-linker penetration and proteome complexity for the data analysis. We followed the reviewer's suggestion and added some sentences in the discussion emphasizing this. [lines 459-465]

What about other features. Did fibril crosslinks occur as well? Can this inform about fiber attachment sites to the capsid?

In theory this approach can detect fibers attached to giant virus capsids and the corresponding attachment site as long as fiber proteins and capsid proteins have lysines in spatial proximity. However, we need to point out that we did not detect such an interaction in this study and we detected comparably few cross-links from the MCP in this dataset. Furthermore, we specifically did not detect cross-links of the MCP to the cap protein on the outside although both are present in high abundance and should have been in contact with the cross-linker. We speculate that the sparsity of cross-links in this region is due to the higher structural integrity of rigid capsid proteins as well as an increased prevalence of modifications (disulfide bridges, glycosylation), which hinder cross-link detection/peptide identification. This would be also true for fiber proteins attached to capsids, as they are also heavily glycosylated.

Reviewer #2 (Remarks to the Author):

In this manuscript, the authors utilized cross-linking MS, quantitative proteomics, and computational biology to characterize the protein architecture of melbournevirus particles. Novel biological insights were generated, including sublayer localization of 88 viral proteins, topological assignment of 25 inner membrane proteins, as well as mapping of 8 proteins in the cryo-EM structure of the capsid. This MS-enabled structural biology study seems thorough and high quality, with consistent results among cross-linking MS, AlphaFold modeling, and viral localization. The scope of the study is also suitable for Nature Communications.

We thank the reviewer for carefully reading and reviewing our manuscript. We are glad to hear the reviewer judges our work as "thorough and high quality" and supports the publication of our manuscript in this journal.

The authors might consider including, but not warranted, validation experiments on protein localization or enzymatic activity assignments, will further strengthen the study. Additionally, this reviewer has some minor suggestions for authors to consider:

1. Authors included less reactive amino acids, such as S, T, and Y, in cross-linked peptide identification, which has the danger to inflate false discovery rate. It will help to include discussion on the prevalence of these less reactive residues in identified peptides.

The reviewer is correct that including S,T and Y as cross-linking sites inflates the search space in the cross-linking search and can pose a challenge to search software. However, it is not true that the false discovery rate is inflated when considering additional XL-sites. This is because the additional search space is not only applied to target sequences but equally applied for decoys. Therefore, adding an infrequent XL-site should not yield a higher rate of false positives but instead fewer overall hits surviving the desired FDR-cutoff. In our case, the addition of S/T/Y for DSSO proved beneficial for data analysis, as it was present at ~8 % (review figure 1).

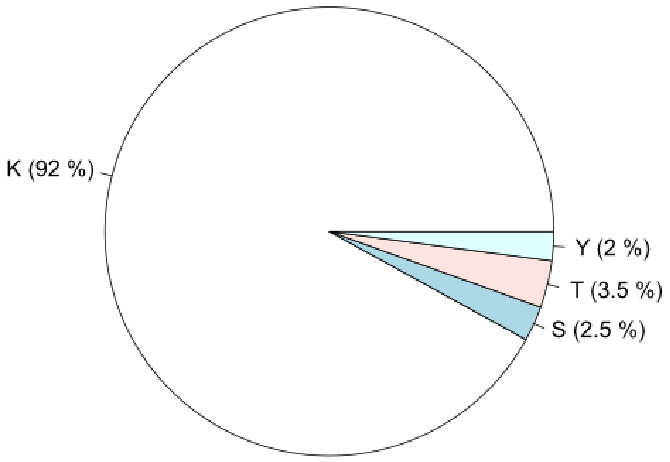

Review Figure 1: fraction of different cross-linked residues at the detected residue-site, derived from the combined cross-linked residue-pair table (Supplementary Table 1, sheet 1).

An FDR inflation resulting from addition of S/T/Y may, however, occur because of improper implementation in XL-MS search software. To avoid such issues, we analyzed our data with the Scout software. Scout is able to consider K/S/T/Y without inflating the FDR, as we have shown previously by benchmarking it against a synthetic ground truth data set (Clasen and Ruwolt et al. NatMethods 2024 https://doi.org/10.1038/s41592-024-02478-1).

2. AlphaFold doesn't have a confidence score of 0-1.0 range. ipTM scores were discussed in the Method section for multimeric prediction. Based on AlphaFold, ipTM scores below 0.6 suggest failed predictions. It will help to clarify the specific confidence scores described in the results.

We apologize for not explaining our quality assessment in enough clarity. For judging the quality of a multimeric prediction we accessed the AlphaFold model confidence score as defined in the AlphaFold multimer pre-print (Evans et al. BioRxiv 2022 https://doi.org/10.1101/2021.10.04.463034, Section 2.8) which is defined as

$$model\ confidence\ =\ 0.2\ *\ pTM\ +\ 0.8\ *\ ipTM$$

We added additional sentences in the methods section to describe this in more clarity. By using a score cut-off of 0.5 we oriented ourselves on other recent studies employing this cut-off on the composite model confidence score or ipTM score to discriminate successful from non-successful predictions (Baptista et al. BioRxiv 2025 https://doi.org/10.1101/2025.06.04.657796, Si and Yan eLife 2024 https://doi.org/10.7554/eLife.92184.2, Banhos Danneskiold-Samsøe et al. CellSys 2024 https://doi.org/10.1016/j.cels.2024.10.004)

3. A total of 16 high confidence models seem low for 88 viral proteins with cross-linking PPIs for sublayer assignment. It will help to discuss possible reasons for such discrepancy, such as the quality of AlphaFold predicted model, or cross-links in unstructured regions etc.

The reviewer has a valid point. However, the impression that 16 out of 88 is comparably low is due to several layers of stringent filtering that we applied to increase the robustness of the reported data that we report below.

Out of 486 PPIs that have been observed in all three replicates (of 88 individual proteins), 475 models could be computed. Of those, 91 had no cross-links in structured regions (pLDDT < 50). Of the remaining 384, 206 models were supported by the cross-linking distance constraint (35 A) for at least half of the cross-links in structured regions. As the reviewer mentioned in the previous point (#2), we also included a confidence score cut-off as a final filter, yielding 16 models. Thus, we included several layers of stringent filtering to provide a set of high accuracy models.

It is true however, that the number of high scoring predictions is relatively low. We largely ascribe this to performance limitations of alphafold multimer on proteins with shallow sequence alignments like viral proteins, especially from novel, uncharacterized viruses. For example, even those minor capsid proteins and their PPIs that we fitted successfully into the cryoEM map had overall poor confidence scores and alphafold multimer failed to predict their correct conformation when in complex (see also our response to Reviewer #4, minor point #4). Furthermore, alphafold multimer usually does not produce high-confidence models when one of the interaction partners has large unstructured regions. A case in point, we identified 7 PPIs involving the core protein MEL_196 (Review Figure 2A). MEL_196's Noumeavirus analog has been shown to undergo liquid-liquid phase separation for initiation of viral factories (Rigou et al. PNAS 2025 https://doi.org/10.1073/pnas.2515074122, see also our response to reviewer #1, point #2 and our revised version of the discussion). Since a

large part of the N-terminus is poorly structured (low pLDDT, Review Figure 2B), no alphafold multimer models succeeded that included this protein.

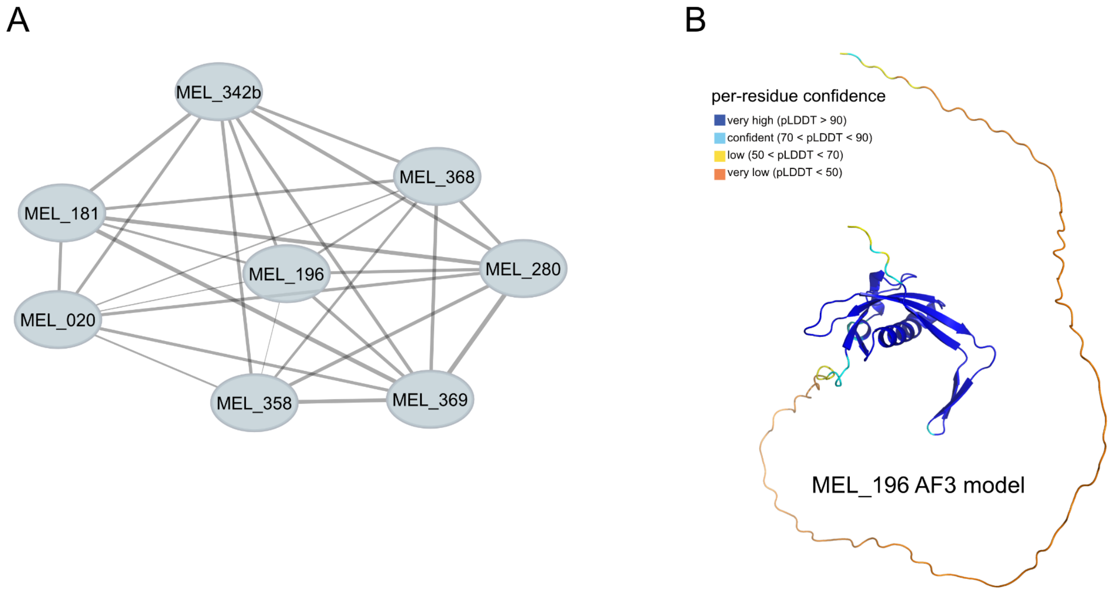

Review Figure 2:

Interactome and model of MEL_196, a core-associated viral protein potentially relevant for initiation of viral factories. A: subnetwork of the core-community of the PPI-map. MEL_196 and all direct interactors are shown with their detected PPIs. B: AF3 model of MEL_196, colored according to pLDDT value.

Reviewer #3 (Remarks to the Author):

I co-reviewed this manuscript with one of the reviewers who provided the listed reports. This is part of the Nature Communications initiative to facilitate training in peer review and to provide appropriate recognition for Early Career Researchers who co-review manuscripts.

We thank the reviewer for carefully reading and co-reviewing our manuscript and are grateful for their comments listed in one of the above reports.

Reviewer #4 (Remarks to the Author):

This work demonstrates the utility of crosslinking mass spectrometry to rapidly provide structural information in poorly understood biological systems, in this case, the giant Melbournevirus. The study provides structural insights using an integrative approach that combines XL-MS with AlphaFold-based modeling to improve our understanding of the architecture of this understudied and complex virus. I am excited to see the use cases of these techniques expanding into different biological samples.

We thank the reviewer for critically reading our manuscript and for the overall positive feedback. We are glad the reviewer judges our project as “exciting”.

However, the impact of this study relies on its addition to the body of knowledge on Melbournevirus biology so the paper needs to be clearer on the structural implications of the discovered structural folds and protein-protein interactions. P

Major concerns:

The authors should include more discussion on the biological implications of the discoveries made in this study.

We extended our discussion in possible biological significance of the findings in the following points:
(i) We expanded the biological context of the finding that predicted enzymes such as glycosyltransferases are located to the coat and nucleic acid related enzymes locate to the core. [lines 391-423]

(ii) we emphasized the scalability to other giant viruses. [lines 459-465]

(iii) we discussed how data relate to latest research findings of initiation of Marseilleviridae replication compartments by viral proteins undergoing liquid-liquid phase separation. [lines 376-382]

The authors use XL-MS as a type of spatial proteomics approach that will indicate which side of the membrane proteins reside on. Why do the authors think that some membrane proteins crosslink to so many proteins on the capsid-facing side of the membrane? Are these non-specific crosslinks?

The reviewer is right. Some coat-domains of viral proteins cross-link to many other proteins in the coat.
The frequency of cross-links for an individual protein is a function of (i) abundance of the protein, (ii) sequence complexity (e.g. how many lysines and tryptic peptides), as well as (iii) structural complexity (e.g. a flexible disordered domain vs a highly ordered and structured domain). This is apparent when looking at MEL_247 (ca. 270 copies), MEL_207 (ca. 1000 copies), and MEL_331 (ca. 450 copies), three highly abundant and most highly connected proteins within the coat domain. Another factor that comes into importance here is the comparably small volume of the coat compartment, as the distance from membrane to capsid is only about 50 to 80 A as observed by cryoEM for the closely related Tokyovirus (Chihara et al., 2022, *Sci reports* https://doi.org/10.1038/s41598-022-24651-2). This compact

space combined with the high stoichiometry of some of the membrane associated proteins explains the extensive identification of cross-linked residue pairs. Please also note that all those interactions were reproducibly detected in three replicates which speaks against non-specific interactions. In addition, our previous work has provided several lines of evidence that the overwhelming majority of detectable cross-links is specific even if intact biological systems are studied (see Liu et al., Mol Cell Proteomics 2018 https://doi.org/10.1074/mcp.RA117.000470, Bogdanow et al., Nat Microbiol 2023 https://doi.org/10.1038/s41564-023-01433-8, Zhu et al., Nat Commun 2024 https://doi.org/10.1038/s41467-024-47569-x)

The section on high-confidence AF3 models with Foldseek leaves the reader wanting much more detail. This section requires some validation of the structurally predicted function of these proteins. In particular, the proposed debiqutinase activity of MEL_206 should be assayed.

To increase the accessibility of these findings, we included two figure panels, indicating the overall frequency of structurally analogous proteins within either coat or core domain, as well as a panel examining the potential catalytic center of MEL_206 with co-predicting the protein with the amoebal ubiquitin variant.

However, it is currently unclear to us which substrates MEL_206 recognizes. For example, it may be specific to specific ubiquitin linkages or to ubiquitin-related proteins like SUMO. Answering these questions would require establishment of protocols for expression and purification of a correctly folded and active MEL_206 followed by thorough in vitro characterization experiments, which we feel is out of the scope of this Manuscript. To begin studying the function of MEL_206, we assayed its ubiquitin affinity through an interaction assay with a vinylsulfone-functionalized HA-tagged human ubiquitin probe, which covalently links to the cysteine residue in the DUB active site. While this workflow enriched several known DUBs from HEK293 cells, enrichment levels from infected amoeba cells were substantially lower (Review Figure 3). No proteins with deubiquitinase function were significantly enriched from infected Acanthamoeba lysates. We speculate that the human ubiquitin probe may not be compatible with the amoeba system, because the amoeba ubiquitin sequence differs in three amino acids. This difference might be enough to abolish human ubiquitin–amoeba DUB interactions, considering the high amino acid specificity of ubiquitin binding to DUBs (Ronau et al. Cell Res 2016 https://doi.org/10.1038/cr.2016.38). This highlights a frequent problem in the work with non-model organisms: specific tools such as amoeba-ubiquitin-vinylsulfone-HA are often not available. Exactly for this reason, integrative workflows like the one we established in our manuscript are valuable to expand biological insights into these systems.

To meet the reviewer's criticism, we toned down some of the sentences regarding the function of MEL_206 and added some sentences expressing the need of additional experiments in the future to substantiate with evidence [lines 295-298].

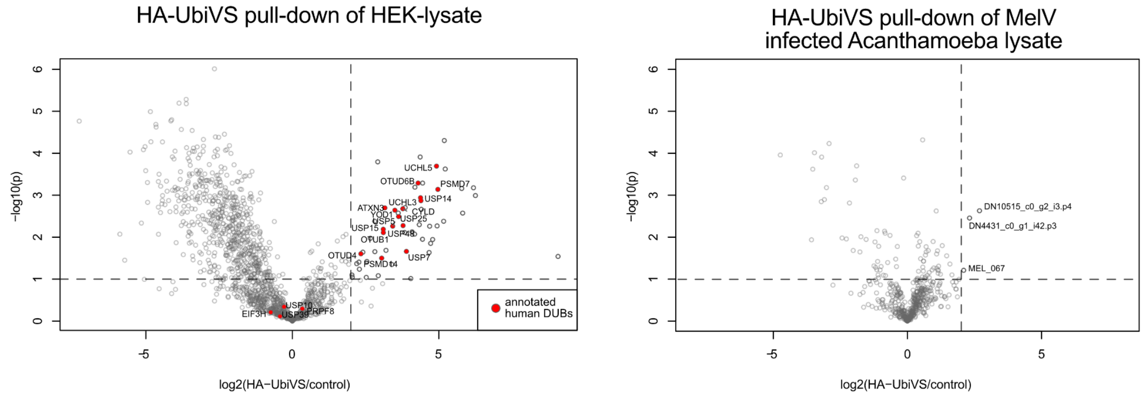

Review Figure 3: Pull-down of Deubiquitinases by HA-tagged Ubiquitin-vinyl-sulfone treatment. Cells were gently lysed in a buffer containing 0.1 % SDS and 1 % NP-40 and several freeze-thaw cycles. Lysate was incubated with 5 mM HA-tagged (human) Ubiquitin vinyl sulfone (HA-UbiVS) which is expected to bind deubiquitinating enzymes. The vinyl-sulfone warhead covalently binds to the catalytic cysteine of DUBs. Non-treated lysates were used as negative control samples. All samples were subjected to HA-immunoprecipitation using the µMACS HA isolation kit (Miltenyi Biotec) as described in Bogdanow et al. Nat Commun 2020 doi.org/10.1038/s41467-020-18542-1. Eluates were digested and subjected to LC-MS measurement as described for LFQ samples in the original manuscript. Every condition was prepared in 3 technical replicates. Fold changes were calculated from LFQ values and missing values were imputed as described before. Volcano plots are built by comparing log2 transformed fold changes against log10 transformed students t-test p-values from the values of three technical replicates. Left: experiment with HEK cell lysate. Annotated DUBs are highlighted in red and are derived from the Epithelial Systems Biology Laboratory (ESBL) website (https://esbl.nhlbi.nih.gov/Databases/KSBP2/Targets/Lists/DUBs/). Many DUBs can be found to be significantly enriched over 8- to 32-fold. Right: experiment with MelV infected Acanthamoeba lysates. Despite experimental conditions being kept the same, nearly no proteins could be found to enrich with HA-UbiVS as efficiently as observed for HEK lysates. Three proteins were enriched over the log2 FC cut-off of 2 (4-fold enrichment): Two acanthamoeba sequences that represent a RNA recognition motif domain containing protein (DN4431_c0_g1_i42.p3) and an unannotated sequence (DN10515_c0_g2_i3.p4), as well as Melbourneviral protein MEL_067, annotated as Translation initiation factor SUI1. None of these proteins have apparent relations to deubiquitinating enzymes. MEL_206 was present in the input but not detected in either of the eluate samples.

The proposed new histone-like viral protein pair MEL_149-MEL_368 is not discussed in enough detail. Does it retain the features of other nucleosome-like structures, such as charged patches for binding DNA?

Yes, the overall arrangement of the complex and specifically the positive surface patches of the alternative Melbournevirus nucleosome resemble known features of other eukaryotic nucleosome-like structures (see below). The positive charged patch is however smaller compared to the MEL-368-MEL_369 complex, which indicates that the DNA-binding might be weaker, and the complex might have more regulative than structural functions like we speculated in the Discussion part of the original manuscript.

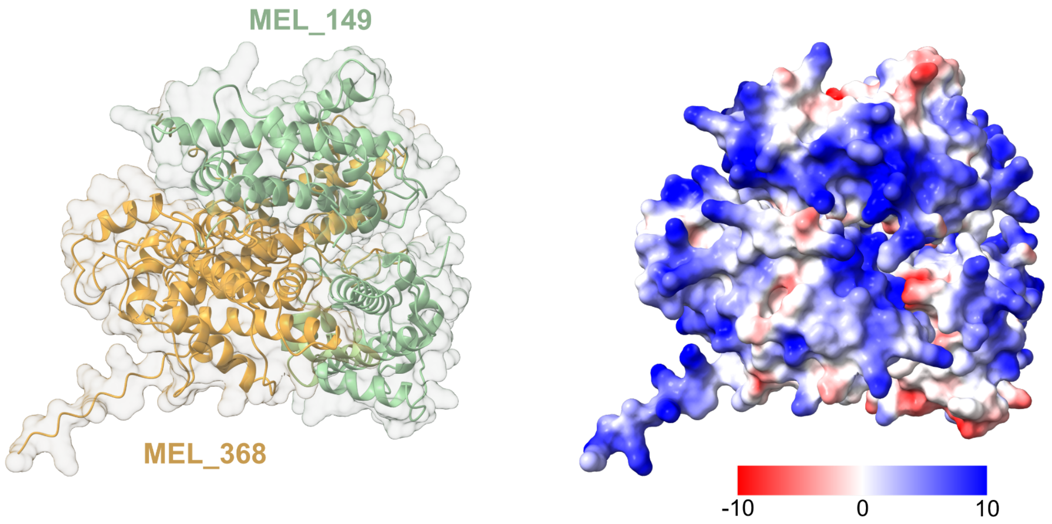

Review Figure 4: AF3 model (left) of the alternative nucleosome-like heterotetrameric MEL_149:MEL_368 complex with the corresponding surface charge (right) computed and plotted in ChimeraX. The surface charge is computed as the coulomb potential in units of kcal/(mol·e) at 298 K.

When our original manuscript was in preparation, the groups of Chantal Abergel and Karolin Luger published a preprint that characterizes this very protein complex in vitro by CryoEM, going into details of structural features. Our data complement this cryoEM study as they provide evidence for the association of those proteins in the native virion. Therefore, we decided against including detailed structural information in our manuscript. Instead, we cited the Abergel/Luger preprint as reference #42 of the original manuscript (now published in Nature Communications and cited as reference #17 of the current manuscript).

Crosslinks have been very often used in the past to help refine model building into cryo-EM densities using crosslinks as distance restraints. The section on expanding the fitted model into the cryo-EM density is interesting, but I wonder if this could have been achieved with AF3 alone using a trial-and-error approach? The crosslinks as distance restraints are not used or shown at all. Do the crosslinks not agree with the suggested interfaces? Can the crosslinks be used to confirm interfaces that are shown and suggest where other proteins should be localised?

We cannot exclude that using brute force an identification of some capsid components using AF3 models alone would be possible. However, there are multiple aspects that made this difficult when we and others tried before: (1) the high number of possibilities for Melbournevirus proteins (403 ORFS, after proteomics ~ 100 proteins in high enough stoichiometry), (2) varying resolution in the CryoEM map with proteins more distant from the vertex being less resolved (see Burton-Smith et al. BioRxiv 2021 https://doi.org/10.1101/2021.07.14.452405), (3) the interweaving nature of the minor capsid proteins makes it hard to decipher where one protein ends and the other begins which makes it hard also to estimate the molecular mass of the proteins, and (4) the comparable low performance of AlphaFold on Melbournevirus proteins. Our integrated strategy made the

identification much easier and straight-forward by capitalizing on the reduction of the candidate list by only looking at proteins in the coat compartment and using interlinks from the major capsid protein, as well as the PCalpha protein. We discussed this strategy and the advantage over trial-and-error in the discussion part extensively.

We did in fact use distance restraints from cross-link data for the allocation of minor capsid proteins and the PC alpha homodimer, as we verified the predicted models with their intra-links. We also attempted using inter-links between identified minor capsid proteins to guide docking. However, since many of the described proteins can appear in alternative complexes outside of the five-fold symmetric capsid vertex (e.g. the two-fold or threefold symmetric center or at the outer area of the cryo-EM map where the resolution gets very low) we did not succeed assigning protein contact sites solely by the cross-linking sites.

Minor comments:

Throughout the manuscript the authors need to be clearer about which metrics they are extracting from AF3 and which they consider to be high confidence. Throughout the manuscript, "confidence score" is used without being defined. Please check every usage throughout the manuscript and improve this in the Methods.

We apologize for not describing our model evaluation in enough clarity. By "confidence score" we meant the composite model confidence score as defined in the AlphaFold multimer pre-print (Evans et al. BioRxiv 2022 https://doi.org/10.1101/2021.10.04.463034, Section 2.8) which is defined as

$$model\ confidence = 0.2 * pTM + 0.8 * ipTM$$

We added additional sentences in the methods section to describe this in more clarity.

Line 202 "supplementary Tab 3" should be "supplementary Table 4"

We corrected the cross reference.

Line 220 "Another insightful prediction is on the viral histone-like proteins. We obtained high-scoring heterodimeric models for MEL_368 (also known as the melbournevirus histone H4-H3-analog) with either MEL_369 (also known as the melbournevirus histone H2B-H2A-analog) or MEL_149 (also known as mini H2B-H2A-analog). While MEL_368:MEL_369 forms canonical nucleosome-like complexes within the virion, MEL_368 and MEL_149 association remained enigmatic 14 and were only recently observed to form a complex in vitro 42."
The crosslinks and AlphaFold models for MEL_368:MEL_369 appear directly comparable to the MEL_368:MEL_149 analysis. Please include the MEL_368:MEL_369 crosslink mapping and model evaluation alongside MEL_368:MEL_149 (e.g., distance-satisfaction statistics, any conflicting links, and a brief structural comparison).

We added a comparison of the heterotetrameric models of MEL_368:MEL_369 and MEL_368:MEL_149 in Supplementary Figure 3. We point out that in contrast to the MEL_149:MEL_368 complex the larger nucleosome complex shows a lot more violated inter-links, even after pLDDT cut-off of 50. This is explainable by the fact that histones when condensing the dsDNA genome stack over each other to compact the DNA in a higher order chromatin structure and this likely represents a mixture of intra-tetrameric links and links from one tetramer to the next one.

Line 281 “However, their lower accuracy domains could not be fitted, likely due to their high flexibility.”
The statement “their lower-accuracy domains could not be fitted, likely due to high flexibility” is reasonable, but you might further test biological context by running AlphaFold 3 with adjacent interactors included in the same input (i.e., co-modeling subsets of the complex). In many cases, AF-Multimer improves interface accuracy when co-evolutionary context is provided. If feasible, please report whether co-modeling improves the problematic domains or interface confidence.

The reviewer is right to suggest predicting multimeric complexes in higher stoichiometry could improve the quality of the prediction. This is of course only possible, when the context of the protein complex is known, e.g. which proteins can be expected to take part of the heteromultimeric complex. In the case of the here mentioned minor capsid proteins MEL_341, MEL_097, MEL_297 and MEL_331 we tried to co-model them with adjacent proteins visible from the cryo-EM map. Specifically, we tried to co-predict MEL_097, MEL_341, two copies of MEL_213b (PCalpha protein) and MEL_234, which were having clear contact sites visible from the cryoEM data. We did not observe an improvement of the protein parts that could not be fitted before. On the contrary, AlphaFold failed to reproduce the observed contact sites of those proteins and confidence scores indicated an unsuccessful prediction (Review Figure 5).

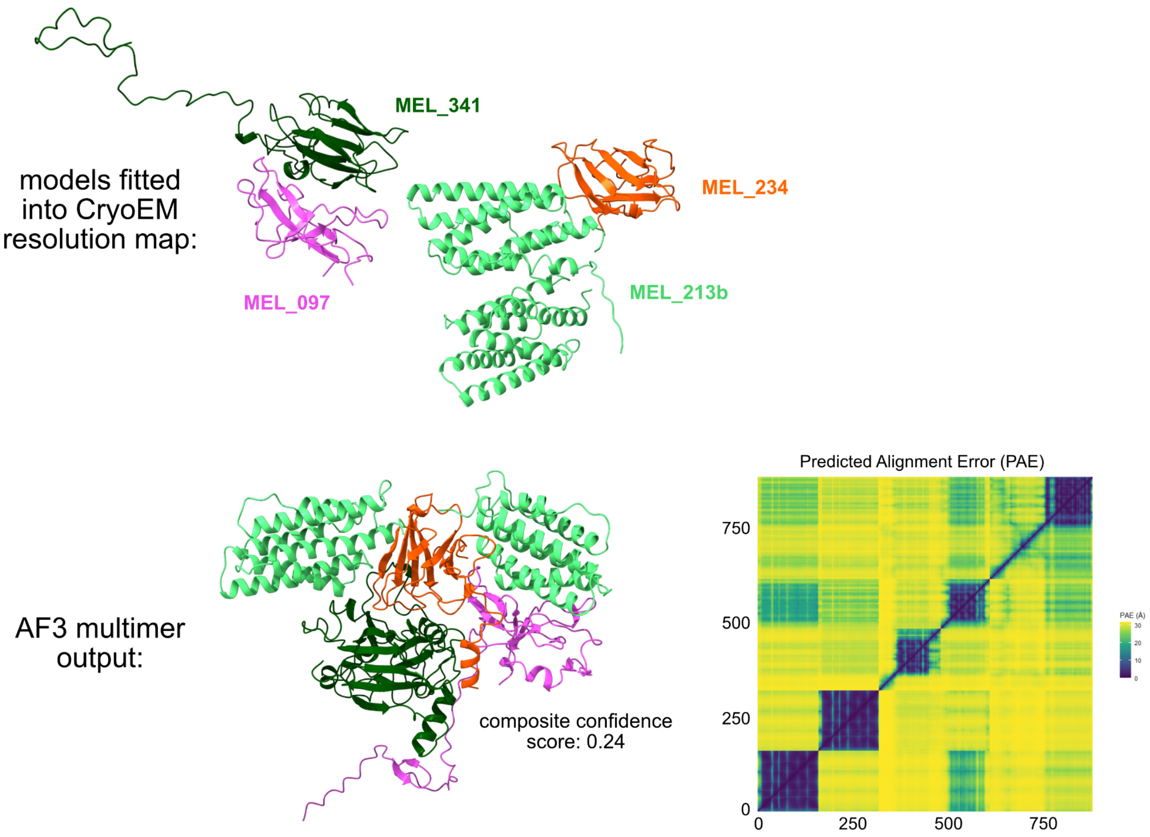

Review Figure 5: Comparison of minor capsid protein complex at the five-fold symmetric capsid vertex with performance of AlphaFold3 multimer, when the whole complex is predicted. Above: complex of minor capsid proteins MEL_341, MEL_097, MEL_213b and MEL_234. Predicted monomer models were fitted into the CryoEM map as described in the manuscript. Only structured and fitted regions are displayed. Below: AlphaFold3 multimeric prediction of all proteins from above. AlphaFold could not reproduce the conformations observed from CryoEM in this prediction. Predicted alignment error (right) as well as the composite confidence score indicated a failed prediction.

Figure 5 For interpretability, please overlay inter-protein crosslinks directly on the structural model in Figure 5 (or add a supplementary panel). Distinguishing inter- vs intra-links visually (e.g., separate colors/line styles) would help readers connect the XL evidence to the proposed interfaces.

We added the information in Supplementary Figure 5. As described in the Results section [lines 328-332], while several inter-protein cross-links are consistent with the fitted interfaces, some discrepancies remain (e.g., the interface between MEL_213b and MEL_276; Supplementary Figure 5). We attribute this to potential protein flexibility or alternative vertex conformations, which have been reported previously for other giant viruses (Shao et al. Nat Commun 2022 https://doi.org/10.1038/s41467-022-34218-4).

Line 298 “Remaining unassigned densities in the cryo-EM map lacked sufficient evidence for confident model matching”
Given the strength of your XL dataset, have you evaluated AlphaLink to help assign proteins to the remaining unmodeled cryo-EM densities?

We thank the reviewer for this suggestion. We are aware that AlphaLink2 can improve structure predictions, especially for structures that exhibit lower ipTM scores (< 0.3) by AlphaFold-Multimer (Gonzalez-Lozano et al. Nature 2025 https://doi.org/10.1038/s41586-025-09059-y). However, in some instances it also yields altered structures without increasing the fraction of satisfied cross-links (Bräuer et al. Commun Chem 2025 https://doi.org/10.1038/s42004-025-01644-6). To test whether integrating the cross-links right into the structure prediction can increase prediction quality of Melbournevirus capsid-associated complexes, we tried AlphaLink2 as well as AF3x (Gilep et al. BioRxiv 2025 https://doi.org/10.1101/2024.12.03.626671), which is an addition to AlphaFold3 utilizing cross-linking reagents as ligands to guide protein complex formation.
We used subsets of cross-links to predict a heteromeric complex formed by a MEL_213b (PC alpha) homodimer and MEL_097 monomer, since this complex is clearly visible from CryoEM and has a good coverage of in-range inter-links (see Reviewer Figure 6). Both AF3x and AlphaLink2 (model weights from AlphaFold2.2) made realistic predictions of the MEL_213b homodimer. However, the quality of their MEL_213b/MEL_097 heterotrimer predictions differed. While AF3x produced realistic models the MEL_213b homodimer within the homotrimer, AlphaLink2 created a new homodimer structure in one instance (Reviewer Figure 6, middle right) and wrongly predicted the orientation of the homodimer interface in another case (Reviewer Figure 6, bottom right). AF3x and AlphaLink2 failed to predict the interface in between MEL_213b and MEL_097 visible in the cryo-EM map. AlphaLink2 might underperform in the present scenario because it was trained on data generated with the Sulfo-SDA crosslinker that imposes a Cα–Cα distance constraint of 25 Å, whereas we crosslinked with DSSO (Cα–Cα distance constraint = 35 Å). Moreover, the heterotrimer might exist in different conformations, which would preclude reconciling the crosslinks into a single correct model. These challenges underscore the importance of the interplay between XL-MS data and experimental structural data in our analysis

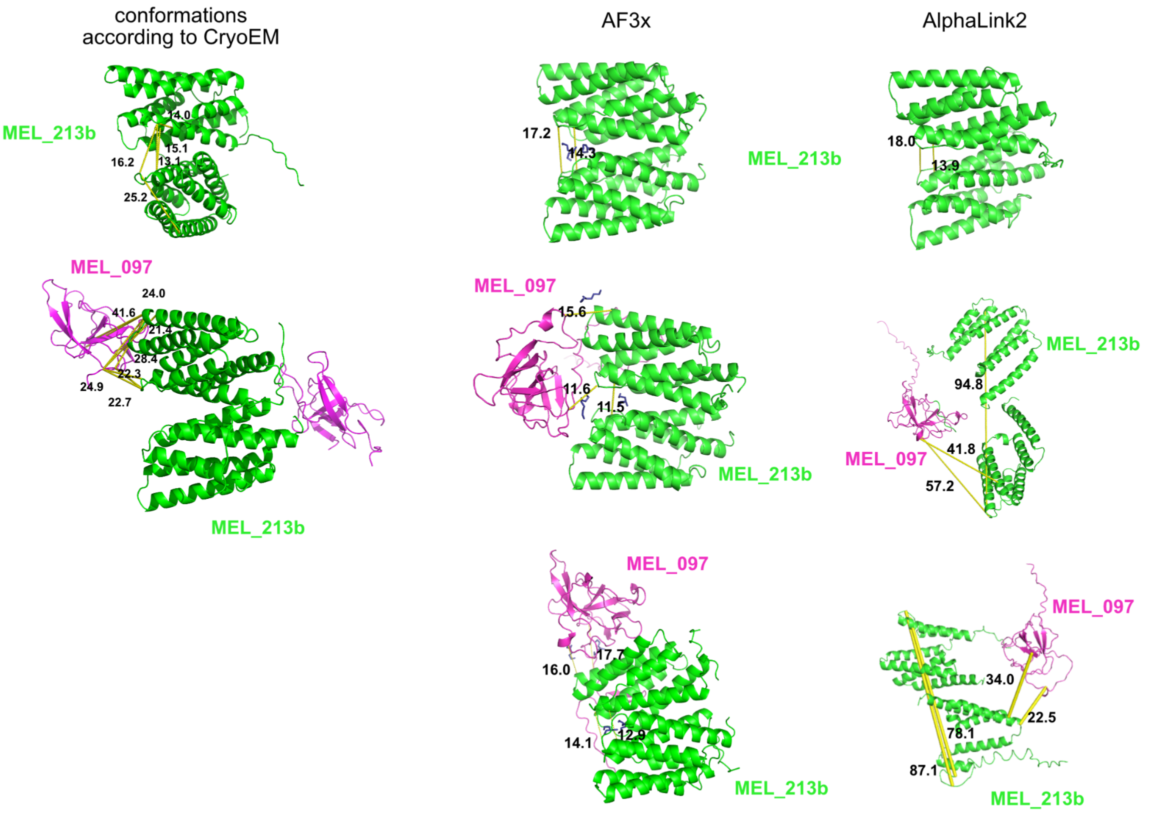

Review Figure 6: Evaluation of performance of AlphaLink2 and AF3X on a selected heteromeric complex of minor capsid proteins MEL_213b and MEL_097. Left: AlphaFold 3 monomer models of MEL_213b and MEL_097 fitted into CryoEM data (according to main Manuscript Figure 5). Only parts that could be fit into the CryoEM map are displayed. Intra-links of MEL_213b subunits (upper) and inter-links between MEL_213b and MEL_097 (lower) are displayed. Center and right: prediction of the MEL_213b homodimer (upper) and MEL_213b-MEL_097 heteromeric complex (middle and lower) by AlphaLink2 (center) and AF3X (right). Different subsets of intra-links or inter-links were used to guide the structure prediction and are displayed.

Reviewer #5 (Remarks to the Author):

I co-reviewed this manuscript with one of the reviewers who provided the listed reports. This is part of the Nature Communications initiative to facilitate training in peer review and to provide appropriate recognition for Early Career Researchers who co-review manuscripts.

We thank the reviewer for carefully reading and co-reviewing our manuscript and are grateful for their comments listed in one of the above reports.

REVIEWERS' COMMENTS

Reviewer #1 (Remarks to the Author):

I am satisfied with the authors' responses to my critiques. Congrats on a lovely methods paper that will be important to the giant virus community.

We thank the reviewer for reviewing our paper and for their positive evaluation.

Reviewer #3 (Remarks to the Author):

I co-reviewed this manuscript with one of the reviewers who provided the listed reports. This is part of the Nature Communications initiative to facilitate training in peer review and to provide appropriate recognition for Early Career Researchers who co-review manuscripts.

We thank the reviewer for co-reviewing our paper and for their positive evaluation.

Reviewer #4 (Remarks to the Author):

The Authors have answered all of our concerns and the manuscript is now suitbale for publishing,

We thank the reviewer for reviewing our paper and for their positive evaluation.
